# The functional role of the structure of the dioxo-isobacteriochlorin in the catalytic site of cytochrome cd_1_ for the reduction of nitrite†
†Electronic supplementary information (ESI) available: Fig. S1–S13. Tables S1–S8. Derivation of eqn (4)–(6). See DOI: 10.1039/c5sc04825g


**DOI:** 10.1039/c5sc04825g

**Published:** 2016-01-20

**Authors:** Hiroshi Fujii, Daisuke Yamaki, Takashi Ogura, Masahiko Hada

**Affiliations:** a Department of Chemistry, Biology and Environmental Science , Faculty of Science , Nara Women's University , Kitauoyanishi , Nara 630-8506 , Japan . Email: fujii@cc.nara-wu.ac.jp; b Department of Chemistry , Graduate School of Science , Tokyo Metropolitan University , 1-1 Minami-Osawa , Hachioji , Tokyo 192-0397 , Japan; c Department of Life Science and Picobiology Institute , Graduate School of Life Science , University of Hyogo , RSC-UH Leading Program Center , 1-1-1 Koto, Sayo-cho, Sayo-gun , Hyogo 679-5148 , Japan

## Abstract

Cytochrome cd_1_ is a key enzyme in bacterial denitrification and catalyzes one-electron reduction of nitrite (NO_2_^–^) to nitric oxide (NO) at the heme d_1_ center under anaerobic conditions. The heme d_1_ has a unique dioxo-isobacteriochlorin structure and is present only in cytochrome cd_1_. To reveal the functional role of the unique heme d_1_ in the catalytic nitrite reduction, we studied effect of the porphyrin macrocycle on each reaction step of the catalytic cycle of cytochrome cd_1_ using synthetic model complexes. The complexes investigated are iron complexes of dioxo-octaethylisobacteriochlorin (**1**), mono-oxo-octaethylchlorin (**2**) and octaethylporphyrin (**3**). We show here that the reduction potential for the transition from the ferric state to the ferrous state and the binding constant for binding of NO_2_^–^ to the ferrous complex increases with a trend of **3** < **2** < **1**. However, the reactivity of the ferrous nitrite complex with protons increases in the reversed order, **1** < **2** < **3**. We also show that the iron bound NO of the ferric NO complex is readily replaced by addition of 1 equiv. of *p*-nitrophenolate. These results indicate that the dioxo-isobacteriochlorin structure is superior to porphyrin and mono-oxo-chlorin structures in the first iron reduction step, the second nitrite binding step, and the NO dissociation step, but inferior in the third nitrite reduction step. These results suggest that the heme d_1_ has evolved as the catalytic site of cytochrome cd_1_ to catalyze the nitrite reduction at the highest possible redox potential while maintaining its catalytic activity.

## Abstract 2

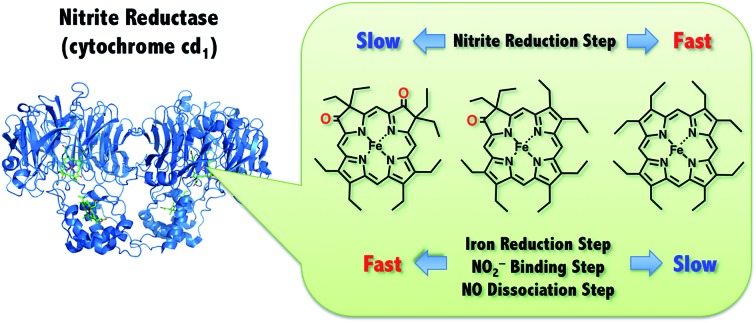
We studied the functional role of the unique heme d_1_ in the catalytic nitrite reduction using synthetic model complexes.

## Introduction

**Fig. 1:**
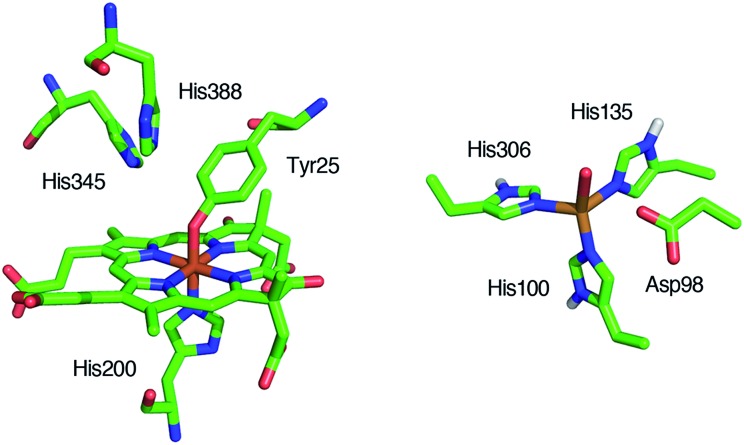
Structures of the catalytic sites of the resting states of cytochrome cd_1_ from *Paracoccus pantotrophus* (left) from PDB file ; 1QKS and Cu-NiR from *Alcaligenes faecalis* (right) from PDB file ; 1AS7.

Reduction of nitrite (NO_2_^–^) to nitric oxide (NO) is an important reaction in the global nitrogen cycle.^1^ This reaction is catalyzed by nitrite reductases in denitrifying bacteria.^1^,^2^ Two types of nitrite reductases exist in nature, copper-containing nitrite reductases (Cu-NiRs) and heme containing nitrite reductases known as cytochromes cd_1_.^2^–^4^ Cytochromes cd_1_ isolated from *Pseudomonas aeruginosa*,^5^–^11^ *Pseudomonas stutzeri*,^12^–^14^ and *Paracoccus pantotrophus*^14^–^20^ have been studied using various methods. Cytochrome cd_1_ is a homodimer, which contains one covalently bound heme c and one noncovalently bound heme d_1_ per monomer.^5^,^9^,^15^,^16^ The heme c assists electron transfer from electron donor proteins, such as cytochrome c_551_ and azurin, to the heme d_1_ site, where NO_2_^–^ is reduced to NO using protons delivered from the distal histidine residues (His-345 and His-388 in *Paracoccus pantotrophus* (Fig. 1)).^9^,^16^ In the oxidized form (the ferric resting state), the phenolic oxygen of the tyrosine residue (Tyr-25) from the heme c domain and the imidazole of the histidine residue (His-200) are coordinated to the heme d_1_.^5^,^9^,^15^,^16^

**Fig. 2:**
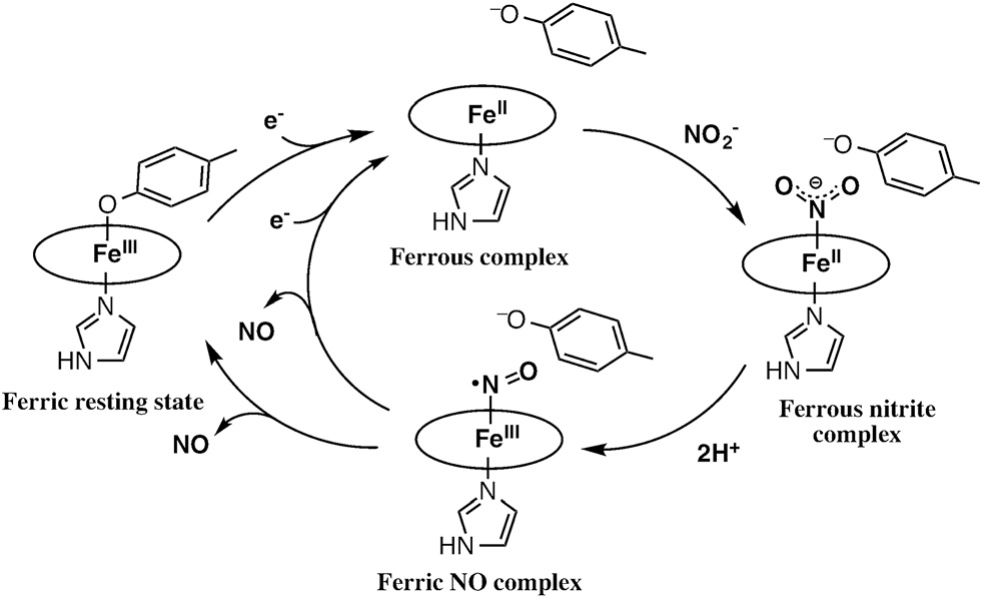
Proposed reaction mechanism of nitrite reduction by cytochrome cd_1_.

The catalytic cycle for the conversion of NO_2_^–^ to NO by cytochrome cd_1_ has been postulated to start from the ferric resting state (Fig. 2).^3^,^5^,^16^ The electron transfer from the electron donors *via* the heme c site reduces the heme d_1_ site from the ferric resting state to the ferrous complex with dissociation of the tyrosine residue, which makes space for the coordination of NO_2_^–^. Then, NO_2_^–^ binds to the ferrous complex to form the ferrous nitrite complex. The electron donation from the ferrous iron center and the proton transfer from the distal histidine residues induce the cleavage of N–O bond of the iron bound NO_2_^–^ to generate the ferric NO complex and water. Finally, the release of the iron bound NO with rebound of the tyrosine residue regenerates the initial ferric resting state. A recent study of Tyr25Ser mutant of *Paracoccus pantotrophus* also implied the participation of the direct pathway from the ferric NO complex to the ferrous complex without the rebound of the tyrosine residue.^21^ In addition, the coordination structures of nitrite to ferric and ferrous porphyrin complexes have been studied by using hemoproteins other than cytochrome cd_1_ and theoretical calculations.^22^–^24^ In contrast, the nitrite reduction of Cu-NiR is catalyzed by two copper ions, which are referred to as the type-I Cu site and the type-II Cu site.^25^–^30^ In Cu-NiR, NO_2_^–^ is reduced to NO at the type-II copper site with copper(i) and copper(ii) redox cycle and an electron required for the nitrite reduction is provided from electron donors, such as azurin, to the type-II copper site *via* the type-I Cu site. It has been proposed that the copper(i) nitrite complex, which corresponds to the ferrous nitrite complex in the catalytic cycle of cytochrome cd_1_, is formed by either NO_2_^–^ binding followed by the electron transfer to the oxidized type-II Cu site, or electron transfer to the oxidized type-II Cu site followed by NO_2_^–^ binding.^29^–^35^ The reaction produces to NO and generates the oxidized type-II Cu site *via* the copper(ii) NO complex with the proton transfer from an aspartate residue. The copper ion in the type-II Cu site forms a complex with three histidine residues (Fig. 1).^25^–^28^

These two types of nitrite reductases have completely different coordination structures in their reaction centers, but catalyze the same reaction. To date, the heme d_1_ exists only in cytochrome cd_1_ and all of the cytochrome cd_1_ enzymes use the heme d_1_ as the catalytic site of nitrite reduction.^36^ Moreover, the three-histidine ligands structure of the type-II site is also common feature in Cu-NiRs.^25^–^28^ These features have led us to question why nitrite reductases utilize these unique reaction centers and have inspired us to investigate the functional role of these active site structures in nitrite reductase activity. Previously, we studied the functional role of the three-histidine ligands of the type-II copper site in Cu-NiR with synthetic type-II Cu site model complexes including sterically hindered triazacyclononane(TACN), tris(imidazolyl)carbinol (TIC) and tris(pyrazolyl)methane (TPM) ligands.^35^ Detailed structural and spectroscopic analysis and theoretical calculations indicated that the TIC ligand has the highest electron donating ability with respect to donation to the copper(i) ion, which increases the nitrite reductase activity. These results led us to propose that strong electron donation from the three-histidine ligands is essential for a high level of Cu-NiR activity. On the other hand, while a previous paper proposed the functional role of the heme d_1_ in cytochrome cd_1_, it has not been revealed how the heme d_1_ controls each reaction step in the catalytic cycle of cytochrome cd_1_.^36^

**Fig. 3:**
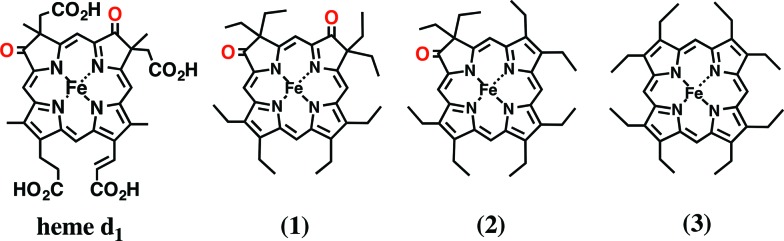
Structures of heme d_1_ in cytochrome cd_1_ and its model complexes used in this study.

In this paper, we investigated the functional roles of the heme d_1_ in nitrite reductase activity with synthetic heme complexes. The heme d_1_ has a dioxo-isobacteriochlorin structure, which contains two saturated pyrrole rings and two electron-withdrawing keto groups in its porphyrin macrocycle. To study the functional role of the heme d_1_ in NiR activity, we prepared model complexes of cytochrome cd_1_ (Fig. 3), iron dioxo-octaethylisobacteriochlorin (**1**), iron monooxo-octaethylchlorin (**2**) and iron octaethylporphyrin (**3**), and quantified the effect of the porphyrin macrocyclic structure on each reaction step of the catalytic cycle of cytochrome cd_1_. The quantification of the porphyrin macrocyle with redox potential, binding constant, and kinetic parameter clearly show that the dioxo-isobacteriochlorin structure of the heme d_1_ is superior to the porphyrin and mono-oxo-chlorin structures in the first iron reduction step from the ferric resting state to the ferrous complex, the second nitrite binding step to the ferrous complex, and the final NO dissociation step from the ferric NO complex. However, the dioxo-isobacteriochlorin structure is inferior in the third nitrite reduction step of the ferrous nitrite complex with protons. Comparison of the present results with the previous results of our Cu-NiR model study allows us to propose an answer to the question of why cytochrome cd_1_ evolved to employ the heme d_1_ and not the more general porphyrin or chlorin, for nitrite reduction.

## Results and discussion

### Ferric resting state model complex

**Fig. 4:**
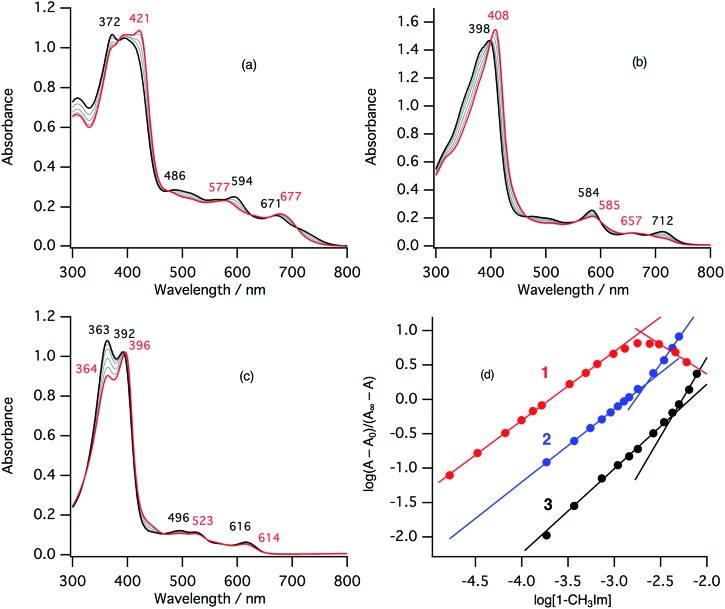
Absorption spectral change for the titration of *p*-nitrophenolate complexes with 1-methylimidazole in acetonitrile at 298 K (a: **1**, b: **2**, c: **3**), and log(*A* – *A*_0_)/(*A*_∞_ – *A*) *vs.* log[1-methylimidazole] plots (d; red: **1**, blue: **2**, black: **3**). The spectra of the *p*-nitrophenolate complexes and the 1-methylimidazole and *p*-nitrophenolate mixed ligand complexes are shown as black and red lines, respectively.

In the ferric resting state of cytochrome cd_1_, histidine imidazole and tyrosine phenolate are coordinated to the ferric heme iron (Fig. 1).^5^,^9^,^15^,^16^ To prepare 1-methylimidazole and *p*-nitrophenolate mixed ligand complex as the ferric resting state model, we initially synthesized ferric mono *p*-nitrophenolate complexes of **1–3**.^37^ The *p*-nitrophenolate complexes were characterized by absorption, ^1^H NMR, and EPR spectroscopies. The binding of *p*-nitrophenolate to the ferric heme centers was confirmed by observation of large alternative paramagnetic shifts of *o*- and *m*-proton signals of the iron bound *p*-nitrophenolate (Fig. S1†). The observed ^1^H NMR shifts were within the range expected for a ferric high spin state.^37^ To make the ferric 1-methylimidazole and *p*-nitrophenolate mixed ligand complexes as the resting state model of cytochrome cd_1_, 1-methylimidazole was titrated into a solution of the *p*-nitrophenolate complexes of **1–3**. Previous ^1^H NMR study reported that 1-methylimidazole binds to *p*-nitrophenolate complexes of ferric porphyrin complexes to form 1-methylimidazole and *p*-nitrophenolate mixed ligand complexes.^38^Fig. 4 shows absorption spectral change for the titration of the *p*-nitrophenolate complexes of **1–3** with 1-methylimidazole. In the first stage of the titration of 1-methylimdazole, the Soret band around 421 nm increases its intensity and the absorption spectrum of the *p*-nitrophenolate complex of **1** changes to a new one (a red line in Fig. 4) having absorption peaks at 395, 421, 577, and 677 nm with clear isosbestic points. Further addition of 1-methylimidazole results in the decrease in the intensity of the Soret band and changes to the absorption spectrum of ferric bis-1-methylimidazole complex of **1** with other clear isosbestic points (Fig. S2†). To analyse the absorption spectral change, plots of log(*A* – *A*_0_)/(*A*_∞_ – *A*) *vs.* log[1-methylimidazole] were constructed, where *A* is the absorbance at the wavelength of interest, *A*_0_ is the absorbance of the initial *p*-nitrophenolate complex, and *A*_∞_ is the absorbance of the 1-methylimidazole adduct (Fig. 4d).^39^ The plot yields slopes of 1.00 and –0.86 for the first and second stages, respectively, indicating the coordination of one equivalent of 1-methylimidazole in each stage. These absorption spectral changes indicate that added 1-methylimidazole binds to the *p*-nitrophenolate complex to form a 1-methylimidazole and *p*-nitrophenolate mixed ligand complex at the first stage, and then the bis-1-methylimidazole complex at the second stage. However, the mixed ligand complex cannot be formed in 100% yield, but at most, 70–80% yield in this conditions. Although the absorption spectrum of cytochrome cd_1_ is the sum of the heme c and heme d_1_ moieties, the absorption spectral features of the heme d_1_ moiety in the range of 600–700 nm are similar to those of the resting state model complex of **1**.^20^ Similarly, 1-methylimidzole is titrated into a solution of the ferric *p*-nitrophenolate complexes of **2** and **3** (Fig. 4b, c and S2†). The slopes in Fig. 4d (1.07 and 1.91 for **2**, and 1.23 and 2.30 for **3**) indicate that **2** and **3** also form bis-1-methylimidazole complexes *via* the 1-methylimidazole and *p*-nitrophenolate mixed ligand complexes, but the maximum yields of the mixed ligand complexes of **2** and **3** are about only 50 and 40%, respectively. The binding constants for the formation of the 1-methylimidazole and *p*-nitrophenolate mixed ligand complexes of **1–3**, estimated from the *y*-intercepts (log *K*) of these plots, are *K* = 5.0 × 10^3^ M^–1^, 1.2 × 10^3^ M^–1^, and 4.8 × 10^2^ M^–1^, respectively. The binding constant increases in the order of **3** < **2** < **1**. This order is the same as that of the binding constant for the formation of bis-imidazole complex reported previously.^40^ Since the binding constant indicates the relative stability of the mixed ligand complex with respect to the initial *p*-nitrophenolate complex, it appears that the dioxo-isobacteriochlorin structure of the heme d_1_ stabilizes the ferric resting state of cytochrome cd_1_.

**Fig. 5:**
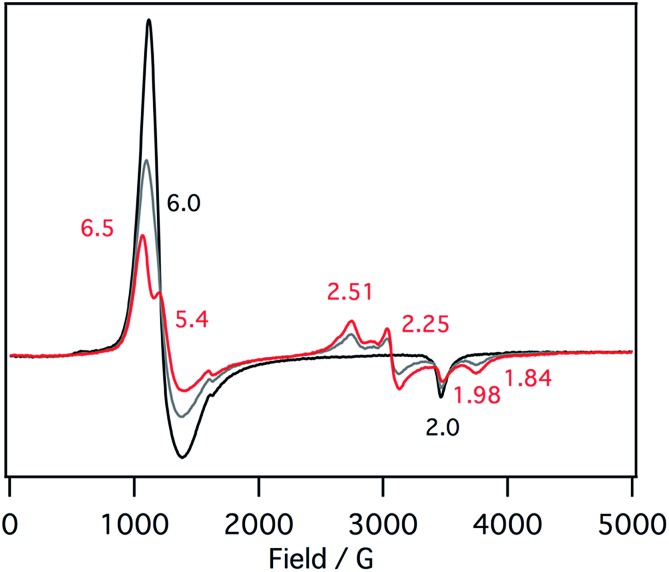
EPR spectral change (4 K) for the titration of the *p*-nitrophenolate complex of **1** with 1-methylimidazole in acetonitrile. Black line: 0 equiv., gray line: 0.5 equiv., red line: 1 equiv.

EPR spectra of the ferric mono *p*-nitrophenolate complexes of **1–3** have signals at *g* = 6.0 and 2.0 and indicate a ferric high spin states in each case (Fig. 5).^37^,^41^ With titration of 1-methylimidazole, the axial EPR spectrum changes to a mixture of a rhombic EPR spectrum with *g* = 6.5, 5.4 and 1.98, typical for ferric high spin species, and a new EPR spectrum with *g* = 2.51, 2.25, 1.84, typical signals for ferric low spin complex. Further addition of 1-methylimidazole affords the EPR spectrum of ferric bis-1-methylimidazole complex at *g* = 2.61, 2.34, and 1.66, as observed in the absorption spectroscopy. These results indicate that the EPR signals observed in the presence of 1 equiv. of 1-methylimidzole are derived from the ferric resting state model complex of **1** and indicate a mixture of ferric high spin and low spin states. A similar EPR spectrum was also reported for a ferric porphyrin complex,^41^ but ferric low spin EPR signals are quite small because of weaker binding of 1-methylimidzolae than an isobactriochlorin complex. In addition, the EPR signals resulting from ferric high state could not be assigned because of the overlap with those of ferric *p*-nitrophenolate complex. These EPR parameters are similar to those of the ferric resting state of the heme d_1_ site of cytochrome cd_1_ from *Paracoccus pantotrophus* (*g* = 6.86, 4.99, and 2.00 for the high spin state and 2.52, 2.19, and 1.84 for the low spin state).^14^

### Reduction to the ferrous complex

The initial step of the nitrite reduction by cytochrome cd_1_ is the reduction of the ferric resting state to the ferrous state (Fig. 2). To investigate the effect of the porphyrin macrocyclic structure on the redox potential of the ferric/ferrous redox potential, we prepared the ferric resting state model complexes (ferric 1-methylimidazole and *p*-nitrophenolate mixed ligand complex) of **1–3** and measured redox potentials of them by cyclic voltammetry (CV) and differential pulse voltammetry (DPV). We could not observe the redox peaks for the ferric resting state model complexes, but observed redox peaks at the potentials similar to those for the *p*-nitrophenolate complex and the bis-1-methylimidazole complex. This is likely due to the difference in the binding constants of 1-methylimidazole between ferric and ferrous porphyrin complexes. Because of extremely large binding constants of the second imidazole to ferrous porphyrin complexes, mono-1-methylimidazole complexes of **1–3** cannot be detected in the titration of 1-methylimidazole to ferrous porphyrin complex. Therefore, the bis-imidazole complex and *p*-nitrophenolate complex are major complexes in the ferrous state. In addition, it is quite reasonable to assume from the binding constants and the redox potentials of heme proteins that the redox potentials of the bis-imidazole complexes are higher than those of the mixed ligand and the *p*-nitrophenolate complexes. Thus, the mixed ligand complexes are reduced to bis-imidazole complexes with its disproportionation to the bis-imidazole complex and *p*-nitrophenolate complex at the redox potentials of the bis-imidazole complexes. As a result, the concentrations of the mixed complexes become lower and the redox peaks for the mixed complexes cannot be detected, as observed in this study. However, we observed positive shift of the redox peak for the *p*-nitrophenolate complex of **1** (from –0.076 V to –0.060 V) with addition of 1-methylimidazole in DPV, probably due to the contribution of the mixed ligand complex. The redox potentials of the *p*-nitrophenolate and bis 1-methylimidazole complexes are listed in Table 1. The redox potentials of the ferric/ferrous redox couple for each of these complexes shifts in the positive direction according to the trend of **3** < **2** < **1**. Previously, Ryan *et al.* reported the redox potentials of ferric chlorin and isobacteriochlorin complexes.^42^ Their data show that this trend results from the electron-withdrawing effect of the keto group. These results suggests that the isobacteriochlorin of the heme d_1_ accelerates the reduction step from the resting state to the ferrous state.

**Table 1:** Redox potentials (V *vs.* SCE) of the ferric/ferrous redox couple for **1–3** in acetonitrile containing 0.1 M tetra-*n*-butylammonium perchlorate^*a*^

	*p*-Nitrophenolate complex	Bis-1-methylimidazole complex	Difference
**1**	–0.076 (∼0.3)	0.097 (0.080)	0.146
**2**	–0.295 (0.135)	–0.135 (0.113)	0.160
**3**	–0.488(0.122)	–0.342 (0.114)	0.173

^*a*^The number in parenthesis are Δ(*E*_p_ – *E*_a_)/volt values.

To characterize the absorption spectrum of ferrous complexes of **1–3**, we performed thin-layer absorption spectroelectrochemistry in acetonitrile containing 0.1 M tetra-*n*-butylammonium perchlorate (TBAClO_4_). By applying more than 100 mV lower voltage than the redox potentials of **1–3**, the absorption spectra of ferric complexes changed to those of ferrous complexes with clear isosbestic points (Fig. S3†). The chemical reduction from ferric model complexes to their ferrous states was also carried out with zinc powder in a glove box. Stirring the ferric complexes of **1–3** with zinc powder in acetonitrile, followed by purification by passing through a silica gel column, provided ferrous complexes of **1–3**, respectively. The absorption spectrum of ferrous complexes of **1** prepared ferric *p*-nitrophenolate complex by zinc reduction was found to be close to that prepared ferric chloride complex, indicating dissociation of *p*-nitrophenolate ligand like the enzyme with reduction (Fig. S4†).

### Binding of nitrite to the ferrous complex

**Fig. 6:**
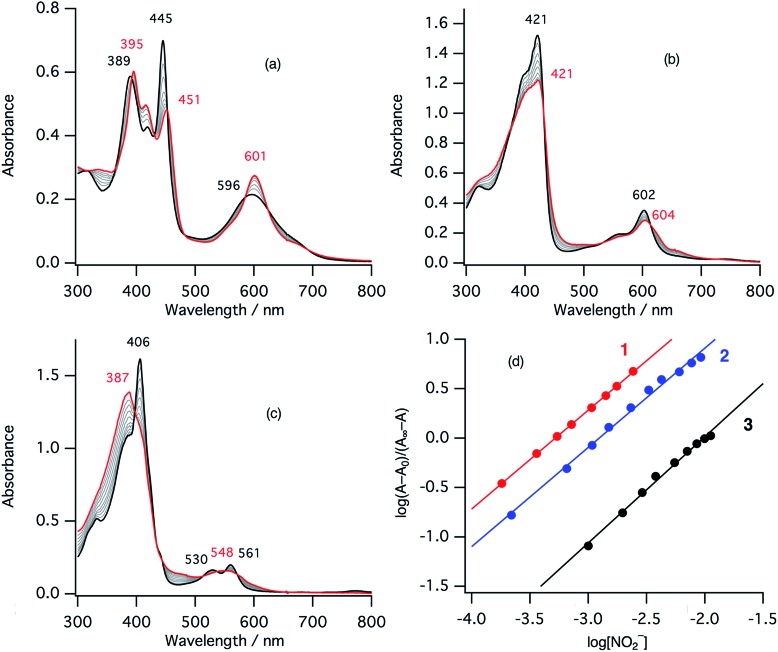
Absorption spectral change for the titration of ferrous complexes of **1–3** with PPN-NO_2_ in acetonitrile at 298 K (a: **1**, b: **2**, c: **3**), and log(*A* – *A*_0_)/(*A*_∞_ – *A*) *vs.* log[NO_2_^–^] plots (d; red: **1**, blue: **2**, black: **3**). The spectra of the ferrous complexes and ferrous nitrite complexes are shown with black and red lines, respectively.

The second step of the nitrite reduction catalyzed by cytochrome cd_1_ is the binding of NO_2_^–^ to the ferrous state (Fig. 2). Structures of ferrous porphyrin nitrite complexes have been reported to have the N-nitro coordination structures of the iron bound NO_2_^–^.^16^,^22^–^24^,^43^–^47^ To study the effect of the porphyrin macrocycle on the nitrite binding step, we examined the reaction of ferrous complexes of **1–3** with bis(triphenylphosphoranylidene) ammonium nitrite (PPN-NO_2_) in acetonitrile. Fig. 6 shows the absorption spectral change for the titration of ferrous complexes of **1–3** with PPN-NO_2_. The absorption spectra of ferrous complexes of **1–3** change to new spectra with clear isosbestic points, indicating binding of NO_2_^–^ to ferrous complexes, with titration of PPN-NO_2_.

To characterize the ferrous nitrite complexes, we analyzed the absorption spectral changes occurring during the titration. Plots of log of the ratio of the nitrite binding form, log(*A* – *A*_0_)/(*A*_∞_ – *A*_0_), *versus* log of NO_2_^–^ were constructed, where *A* is the absorbance at the wavelength of interest, *A*_0_ is the absorbance in the absence of nitrite ion, and *A*_∞_ is the absorbance in the presence of a large excess of nitrite (Fig. 6d).^39^ The slopes of the plots, which indicate the number of nitrite ligands bound to ferrous heme, were 1.00 for **1**, 1.00 for **2**, and 1.08 for **3**. These results indicate that, in each case, only one nitrite ligand binds to the ferrous heme to form a five-coordinate mono nitrite complex. The *y*-intercepts of the plots, which indicate log of the binding constants (log *K*), were 3.29 (*K* = 2.5 × 10^3^ M^–1^) for **1**, 2.93 (*K* = 8.3 × 10^2^ M^–1^) for **2**, and 2.16 (*K* = 1.4 × 10^2^ M^–1^) for **3**. The binding constant of NO_2_^–^ to the ferrous heme complex increases according to the trend of **3** < **2** < **1**. To further confirm the binding of NO_2_^–^, ESI-mass spectrometry (ESI-MS) were carried out for the complexes. The negative ESI-MS spectra of the reaction products of **1**, **2**, and **3** with NO_2_^–^ show major signals at *m*/*z* 666.48, 650.55 and 634.56, respectively (Fig. S3†). These signals shift by one mass unit when ^15^N-labeled nitrite (^15^NO_2_^–^) is used (Fig. S3†). The mass numbers and their isotope patterns are fully consistent with five-coordinate mono nitrite complexes.

We also studied effect of the axial imidazole ligand on the nitrite binding to more mimic the ferrous nitrite complex of cytochrome cd_1_. The titration of nitrite to ferrous complex of **1** in the presence of 1 equiv. of 1-methylimidazole and 50 equiv. of 1,2-dimethylimidazole showed absorption spectral change to new spectra close to ferrous nitrite complex of **1** with clear isosbestic points (Fig. S6†). The absorption spectra of ferrous nitrite complexes in the presence of these imidazole ligands are similar to that in the absence of these imidazole ligands. The titration experiments showed that 1 equiv. of nitrite binds to ferrous complexes and the binding constants of nitrite in these conditions were estimated to be log *K* = 3.04 and 3.15 for 1-methylimidazole and 1,2-dimethylimidazole, respectively, which are slightly lower than that in the absence of imidazole ligand.

**Fig. 7:**
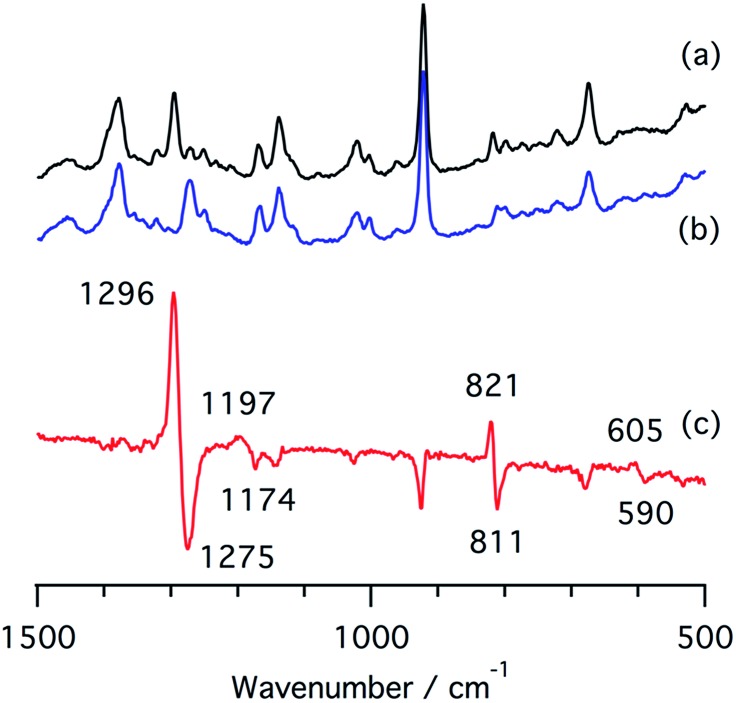
Resonance Raman spectra of (a) a ferrous nitrite complex of **1**, (b) a ^15^N-labelled ferrous nitrite complex of **1**, and (c) their difference spectrum, (a and b).

We also measured resonance Raman spectra of the ferrous nitrite complex of **1** (Fig. 7). We found isotope sensitive bands at 1296, 1197, and 821 cm^–1^, which shift to 1275, 1174, and 811 cm^–1^ when ^15^N-labiled nitrite, PPN-^15^NO_2_, is used. These vibrational energies are close to those of copper nitrite complexes.^35^,^48^ These bands are tentatively assigned as the symmetric O–N–O stretching band, the asymmetric O–N–O stretching band, and the scissor mode on the basis of their intensities and isotope shifts.

### Reaction of the ferrous nitrite complex with protons

**Fig. 8:**
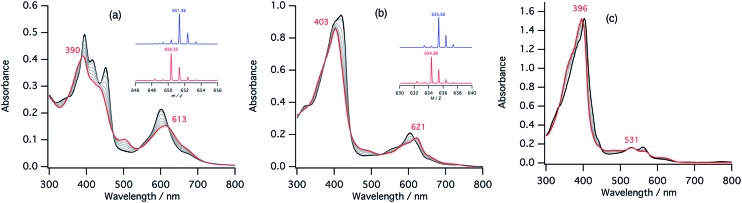
The absorption spectral change for the reaction of ferrous nitrite complexes of **1–3** with acetic acid in acetonitrile at 298 K. (a) **1** = ∼7.9 μM, [NO_2_^–^] = 3.92 mM, [CH_3_COOH] = 1.12 mM, time interval 90 s, (b) **2** = ∼7.9 μM, [NO_2_^–^] = 3.92 mM, [CH_3_COOH] = 1.12 mM, time interval 15 s, (c) **3** = ∼10 μM, [NO_2_^–^] = 19.8 mM, [CH_3_COOH] = 1.12 mM, time interval 45 s. The absorption spectra of the initial solution and final solution are shown with black and red lines, respectively. Inset; ESI-MS (positive) spectra of the final reaction solution for ferrous nitrite complexes (red lines) and ^15^N-labelled ferrous nitrite complexes (blue lines).

The ferrous nitrite complex is reduced to the ferric nitric oxide complex by the reaction with protons in the catalytic cycle of cytochrome cd_1_ (Fig. 2). We examined the reaction of ferrous nitrite complexes of **1–3** with acetic acid. Fig. 8 shows absorption spectral changes occurring in the reaction of ferrous nitrite complex of **1** with acetic acid. The absorption spectrum of the ferrous nitrite complex converts to a new spectrum with absorption peaks at 390 and 613 nm with clear isosbestic points. The final spectrum is similar to that of the nitric oxide complex of **1**.^40^ The formation of the ferric nitric oxide complex was confirmed by ESI-MS measurements. The positive ESI-MS spectrum of the final reaction solution has a mono cation peak at 650.35. The peak position and isotope pattern are consistent with a mono cation peak corresponding to a ferric nitric oxide complex, [**1**-NO]^+^. In addition, this peak shifts by one mass unit when ^15^N labeled nitrite is used (Fig. 8). These results indicate that the ferrous nitrite complex of **1** reacts with acetic acid to form the ferric nitric oxide complex. Since excess NO_2_^–^ is present in the reaction solution, NO_2_^–^ may be also bound to the ferric nitric oxide complex. Ferrous nitrite complexes of **2** and **3** also react with acetic acid. With addition of acetic acid, the absorption spectra of ferrous nitrite complexes of **2** and **3** each change to the spectra similar to those of nitric oxide complexes of **2** and **3**, respectively with clear isosbestic points (Fig. 8).^40^ The ESI-MS measurements and ^15^N-labeling experiments also provide evidence in support of the formation of the ferric nitric oxide complexes of **2**. However, the ESI-MS data for the ferric nitric oxide complexes of **3** could not be obtained. The ferric nitric oxide complex of **3** seems to be less stable than that of **1**. However, experimentally, it is believed that heme d_1_ forms a less stable nitric oxide complex than the normal hemes.^15^,^16^ This study shows that the binding of *p*-nitrophenolate stabilizes the ferric resting state model complex of **1** much more than **3**, thus, the tyrosine residue in the active site would relate to the instability of the ferric nitric oxide complex in the enzyme.

To compare the nitrite reduction activity between ferrous nitrite complexes of **1–3**, we estimated the reaction rate constants for the reactions of ferrous nitrite complexes with acetic acid in CH_2_Cl_2_ at 295 K. Under the pseudo first-order conditions (in the presence of excess NO_2_^–^ and acetic acid relative to **1–3**), the time courses of the change in absorbance occurring during the reactions could be fit with single exponential functions, providing apparent reaction rate constants. When the concentration of NO_2_^–^ was increased under a constant concentration of acetic acid, the apparent reaction rate constants for **1–3** were found to increase linearly in the low NO_2_^–^ concentration region before attaining relatively constant values at high NO_2_^–^ concentrations (Fig. S7†). We could not determine the reaction rate constant for **3** at high NO_2_^–^ concentrations because the reaction was too fast to determine the rate constant with an absorption spectrometer. The observed kinetic change can be explained by the following scheme.

1 (P)Fe^II^ + NO_2_^–^ ⇄ [(P)Fe^II^–NO_2_]^–^

2 [(P)Fe^II^–NO_2_]^–^ + CH_3_CO_2_H ⇄ [(P)Fe^III^–NO_2_H](CH_3_CO_2_)

3 [(P)Fe^III^–NO_2_H](CH_3_CO_2_) + CH_3_CO_2_H → (P)Fe^III^–NO + H_2_O + 2CH_3_CO_2_^–^

The reaction step shown in eqn (2) is the rate-limiting step of the overall reaction because the absorption spectral changes and the presence of clear isosbestic points shown in Fig. 8 indicates the first protonation step is the slowest reaction. Therefore, when we introduce steady-state approximation for [(P)Fe^III^–NO_2_H](CH_3_CO_2_), the reaction rate would be as follows.4
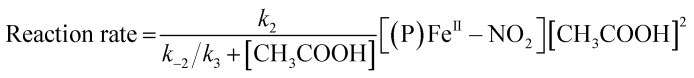
where [(P)Fe^II^–NO_2_] is the concentration of the ferrous nitrite complex, [CH_3_COOH] is the concentration of acetic acid, *k*_2_ is the reaction rate of the forward reaction of eqn (2), *k*_–2_ is that of the reverse reaction of eqn (2), and *k*_3_ is reaction rate of eqn (3) (details are shown in SI.).

**Fig. 9:**
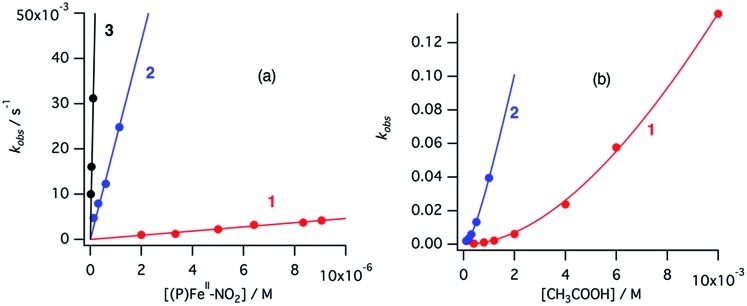
Dependence of apparent reaction rate constant (a) on the concentration of [(P)Fe^II^–NO_2_]^–^ at constant acetic acid concentration, [CH_3_COOH] = 1.12 mM, and (b) on the concentration of acetic acid at constant nitrite concentration, [NO_2_^–^] = 3.92 mM for **1** and [NO_2_^–^] = 1.96 mM for **2** in acetonitrile at 298 K. The red, blue, and black indicate data for **1**, **2**, and **3**, respectively.

Under the constant concentration of acetic acid, eqn (4) can be further simplified with concentration of NO_2_^–^ ([NO_2_^–^]) and the equilibrium constant (K) for eqn (1), as shown eqn (5).5
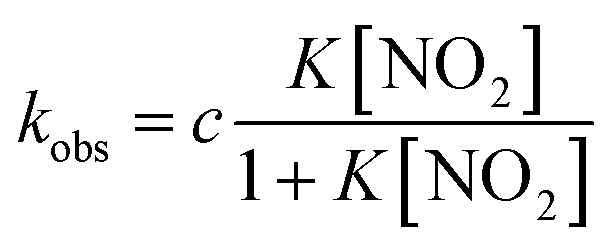
where *c* is a constant including *k*_2_, *k*_–2_, *k*_3_ and [CH_3_CO_2_H] values. We could effectively simulate well the saturation behavior observed for **1** and **2** with eqn (5) and the *K* values estimated from the titration experiments (Fig. S7†). To compare the reactivity between **1–3** in detail, we repeated the kinetic measurements at a constant concentration of acetic acid and plotted the apparent reaction rate constant against the concentration of [(P)Fe^II^–NO_2_]^–^ calculated from the estimated *K* values (Fig. 9). Since the apparent reaction rate would be proportional to the concentration of [(P)Fe^II^–NO_2_]^–^, as shown in eqn (4), the gradients of the plots indicate the reactivity of the ferrous nitrite complex with acetic acid. The reactivity of the ferrous nitrite complex increases according to the order of **1** < **2** < **3**.

Similarly, under the constant concentration of NO_2_^–^, the apparent reaction rate increases with increasing concentrations of acetic acid. However, the plots for **1** and **2** provide curved lines instead of a linear relationship (Fig. 9). Similar non-linear behavior has been observed for the reaction of copper(i) nitrite complexes with acetic acid and could be analyzed well with eqn (6), which could be easily derived from eqn (4).^35^ We attempted to simulate the dependence of *k*_obs_ with eqn (6).6
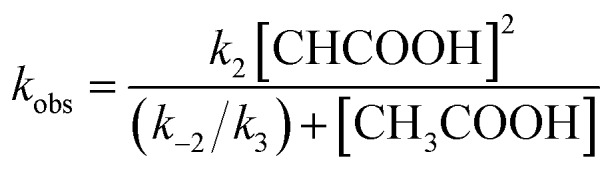



According to this equation, it can be expected that *k*_obs_ would be proportional to the concentration of acetic acid if *k*_3_ is much larger than *k*_–2_, (*k*_3_ ≫ *k*_–2_: *k*_–2_/*k*_3_ ≈ 0) while the dependence of *k*_obs_ would be curved when *k*_–2_/*k*_3_ is comparable to the concentration of acetic acid. As shown in Fig. 9, the dependence of *k*_obs_ on the concentration of acetic acid fit well with eqn (6) and the estimated *k*_2_ and *k*_–2_*/k*_3_ values are summarized in Table 2. The *k*_2_ and *k*_–2_*/k*_3_ values are changed by the porphyrin macrocyclic structure. Although **1** is the least reactive of the three porphyrin complexes, the estimated *k*_2_ and *k*_–2_*/k*_3_ values for **1** are similar to those of the most reactive copper(i) nitrite complex, (Et-TIC)Cu(NO_2_), reported previously. Because of this high reactivity, reliable *k*_2_ and *k*_–2_*/k*_3_ values for **3** could not be obtained, suggesting that the *k*_2_ value is much larger and the *k*_–2_*/k*_3_ value is much smaller.

**Table 2:** Reaction rate constants for reactions of nitrite complexes with acetic acid

	**1** ^*a*^	**2** ^*a*^	(Et-TIC) Cu (NO_2_)^*b*^	(iPr-TACN) Cu (NO_2_)^*b*^	(Et-TPM) Cu (NO_2_)^*b*^
*k*_2_ (M^–1^ s^–1^)	51.5 (56.7)^*b*^	70.6 (115.0)^*b*^	47.9	2.08	0.41
*k*_–2_/*k*_3_ (M)	2.7 × 10^–2^	7.9 × 10^–4^	∼0	2.0 × 10^–2^	6.2 × 10^–2^

^*a*^[NO_2_^–^] = 3.92 mM for **1** and [NO_2_^–^] = 1.96 mM for **2** in acetonitrile at 298 K. The values in the parenthesis are estimated values for pure ferric nitrite complexes (for the conditions including a large excess of nitrite).

^*b*^Et-TIC = tris(1-methyl-2-ethyl-4-imidazolyl)carbinol, iPr-TACN = 1,4,7-triisopropyl-1,4,7-triazacyclononane, Et-TPM = tris(3,5-diethyl-1-pyrazolyl)methane. The rate constants for these copper complexes are cited from ref. 35 at 293 K.

### MO calculations

**Fig. 10:**
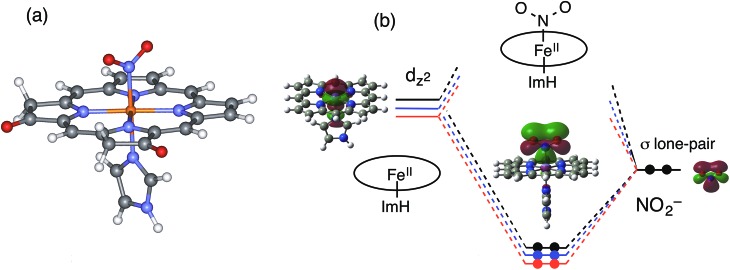
Optimized model structure of the ferrous nitrite complex for the structurally simplified **1** (a) and molecular orbital interactions for the formation of Fe–N(nitrite) bonds of **1–3** (b). Red line: **1**, blue line: **2**, and black line: **3**.

To gain further insights into the electronic structure and reactivity of the ferrous nitrite complex, molecular orbital (MO) calculations were carried out for the ferrous nitrite complexes and the ferrous complexes of **1–3**. The ethyl substituents in **1–3** were replaced with hydrogen (H) to simplify the systems for calculations and imidazole was coordinated as an axial ligand to mimic the heme d_1_ site of cytochrome cd_1_. According to the crystal structures,^14^,^43^–^46^ nitrite was coordinated to the ferrous iron with N-nitro binding form. The optimized structures, their selected bond parameters, and total energies for the ferrous nitrite complexes are shown in Fig. 10, S8 and Tables S1, S2.† The Fe–N(nitrite) bond length becomes shorter in the order of **3**, **2**, and **1**, suggesting that the binding of nitrite to the ferrous iron center becomes stronger according to this trend. This is further confirmed by the calculated binding energies of the ferrous complexes to nitrite; the binding energies of **1** and **2** are 54.0 kJ mol^–1^ and 26.8 kJ mol^–1^ greater than that of **3**, respectively (Table S2†). The N–O bond distance of the iron-bound nitrite is shorter than that of free nitrite and also becomes shorter in the same order as the Fe–N(nitrite) bond length (**3**, **2**, and **1**). On the other hand, the Fe–N(Por) bond length becomes shorter in the order of **1**, **2**, and **3**, suggesting that the binding of the porphyrin ligand becomes stronger according to this trend.

To explain the altered reactivity change, we prepared the orbital energy diagrams for the ferrous nitrite complexes (Fig. 10 and S9†). These orbital energies are listed in Tables S3–S5.† With coordination of nitrite, the spin state of ferrous iron changes from high-spin to low-spin, consistent with the experimental results.^43^–^46^,^48^ The Fe–N(nitrite) bond for the ferrous nitrite complex consists of an interaction between the iron d_*z*_^2^ orbital of the ferrous complex (unoccupied orbital for the ferrous low-spin state) and the occupied p_σ_ orbital of nitrite (the HOMO of free nitrite). Therefore, the strength of the Fe–N(nitrite) bond is determined by the energy of the iron d_*z*_^2^ orbital of the ferrous complex. As the energy of the iron d_*z*_^2^ orbital is lower, the Fe–N(nitrite) bond becomes stronger and electron transfers from the iron bound nitrite to the iron side is more effective.

In this study, we examined the experiments for ferrous complexes in acetonitrile. To further investigate axial ligand effect on the structure and orbital energy, we performed DFT calculations for ferrous complexes and ferrous nitrite complexes of **1–3**, as well as those having acetonitrile axial ligands. The results are summarized in Fig. S10–S13 and Tables S6–S8.† The calculations indicated that the ferrous complexes having acetonitrile axial ligands are more stable than those without the axial ligands. The main conclusions from the DFT calculations are not changed even when the axial imidazole ligands in the model complexes are removed or replaced with acetonitrile solvent molecule. The binding of nitrite becomes stronger in the order of **3**, **2**, and **1**, and the 3d_*z*_^2^ orbital of the ferrous complexes and the HOMO of the ferrous nitrite complexes are stabilized in the same order (**3**, **2**, and **1**).

Why is the iron d_*z*_^2^ orbital of the ferrous complex stabilized in the order of **3**, **2**, and **1**? Since the iron d_*z*_^2^ orbital of the ferrous complex interacts with the lone pair σ-donor orbital of the pyrrole N atom of the porphyrin macrocycle, the energy of the iron d_*z*_^2^ orbital should be controlled by the energy of the lone pair σ-donor orbital of the pyrrole N atom. The energy of the iron d_*z*_^2^ orbital of the ferrous complex becomes higher as the energy of the σ-donor orbital of the pyrrole N atom is higher and the interaction becomes stronger. Since the electron-withdrawing keto group stabilizes the σ-donor orbital of the pyrrole N atom, the energy of the iron d_*z*_^2^ orbital of the ferrous complex is stabilized as the number of the keto groups added to the porphyrin macrocycle increases according to the trend of **3** < **2** < **1**, (Fig. 10).

In the previous Cu-NiR study, we reported that the reactivity of copper(i) nitrite complex with protons increases with an increase in the energy of its HOMO, which consists of the interaction of the copper 4s orbital with the occupied p_σ_ orbital of nitrite and has electron density mainly in the copper-bound nitrite.^35^ The reactivity of the ferrous nitrite complex with protons is also explained by the energy of the similar orbital, the bonding orbital between the iron d_*z*_^2^ orbital and the occupied p_σ_ orbital of nitrite. This orbital also has electron density mainly in the iron bound nitrite (Fig. 10). As discussed above, the electron-withdrawing keto group in the porphyrin macrocycle leads to stabilization of the σ-donor orbital of the pyrrole N atom, which, in turn, causes a decrease in the energy of the iron d_*z*_^2^ orbital and stabilization of the bonding orbital between the iron d_*z*_^2^ orbital and the occupied p_σ_ orbital of nitrite (Fig. 10). Since this bonding orbital interacts with a proton, the protonation reaction of the ferrous nitrite complex becomes slower as the number of keto groups in porphyrin macrocycle increases.

### Replacement of NO with rebinding of phenolate

The final reaction step of nitrite reduction by cytochrome cd_1_ is replacement of the heme bound NO with a tyrosine residue to form the ferric resting state (Fig. 2). Previously, this reaction step has been studied with 1-methylimidazole.^40^ Addition of 1-methylimidazole to the ferric NO-1-methylimidazole complexes of **1–3** was found to form corresponding ferric bis-1-methylimidazole complexes with ligand exchange. The equilibrium constant of the ligand exchange reaction increased in the order of **3** < **2** < **1**, indicating that **1**, which is the most stable NO complex in these three complexes, is the easiest complex to exchange the iron-bound NO with 1-methylimidazole. This can be explained by the change of the reaction free energy of the NO dissociation reaction. Since the binding of NO to ferric complex is much weaker than that of 1-methylimidazole, the reaction free energy is mainly dominated by the stability of the bis-1-methylimidazole complex. The binding constant of 1-methylimidazole increases in the order of **3** < **2** < **1**, resulting in the easiest dissociation of NO from the most stable NO complex, **1**. The stability of the ferric resting state seems to control the final NO dissociation step in the catalytic cycle.

Here, to better mimic the cytochrome cd_1_ reaction, we performed a titration of ferric NO-1-methylimidazole complex of **1** with tetra-*n*-butylammonium *p*-nitrophenolate (TBA-PhO). The absorption spectrum of the ferric NO-1-methylimidazole complex of **1** changes to that of the ferric *p*-nitrophenolate complex with addition of 1 equiv. of TBA-PhO (Fig. S10†). This result indicates that, as the final step of the cytochrome cd_1_ reaction, the heme bound NO is replaced with addition of only1 equiv. of *p*-nitrophenolate ligand. Addition of *p*-nitrophenolate ligand initially forms the ferric resting state model complex with NO dissociation, but 1-methylimidazole dissociates from the ferric resting state model complex because of the low affinity of 1-methylimidazole to the ferric *p*-nitrophenolate complex as shown in this study.

### Functional role of heme d_1_

In this last section, we would like to discuss on the functional role of the heme d_1_ in nitrite reductase on the basis of the present results. This study indicates that the redox potential shows positive shift in the order of **3** < **2** < **1** and the redox potential of **1** is close to those of the electron donor proteins such as cytochrome c_551_ and azurin (∼+10 mV *vs.* SCE).^49^ The electron donors can reduce the ferric resting state if cytochrome cd_1_ has **1** as a catalytic center. However, the electron donor proteins would encounter difficulties in reducing the ferric resting state to the ferrous complex if cytochrome cd_1_ was employed **3** as the catalytic center although it is known that the redox potential of a heme active site in a heme protein is influenced by the characteristics of its axial ligand and the protein environment of the heme. In fact, the cytochrome cd_1_ reconstituted protohemin (ferric complex of protoporphyrin IX) has been reported to have no nitrite reductase activity.^10^ The dioxo-isobacteriochlorin structure of the heme d_1_ enhances electron transfer for nitrite reduction from the electron donor proteins. The dioxo-isobacteriochlorin structure is also superior for the next nitrite-binding step. Since the binding constant of nitrite to the ferrous complex increases in the order of **3** < **2** < **1**, the dioxo-isobacteriochlorin structure of the heme d_1_ is effective in binding nitrite. The present MO calculations reveal that the high affinity of the ferrous complex of **1** for nitrite is a result of low electron density of the iron center due to the strong electron-withdrawing effect of the two dioxo groups in **1**. This property would be important for denitrifying bacteria when the concentration of nitrite in the bacteria is low. On the other hand, this study shows that the reactivity of the ferrous nitrite complex is increased in the order of **1** < **2** < **3**, which is the opposite to the trend for redox potential and the trend for nitrite binding. The dioxo-isobacteriochlorin structure of heme d_1_ would not be more favorable for nitrite reduction than the porphyrin and mono-oxo chlorin structures. The lowest electron density of the iron center of **1** decreases the p*K*_a_ value of the iron-bound nitrite and slows the protonation step of the iron-bound nitrite, resulting in the lowest reactivity. However, even **1** would have enough activity to be effective as a nitrite reductase because the kinetic data of **1** is comparable to that of the most reactive copper nitrite reductase model complex, (Et-TIC)Cu(NO_2_).^40^ It has been proposed that the NO dissociation in the final step is accelerated by the dioxo-isobacteriochlorin ligand. This is confirmed by the present finding that the iron bound NO of the ferric NO complex of **1** is readily replaced by only 1 equiv. of *p*-nitrophenolate ligand. Since the electron transfer from electron donors to the ferric NO complex before NO dissociation results in inhibition of the catalytic cycle due to the formation of a stable ferrous NO complex, rapid dissociation of NO from the ferric NO complex is essential to retain the nitrite reductase activity.

The nitrite reduction reaction is one step of the denitrification process, which relates to the ATP synthesis under anaerobic conditions. Therefore it is essential for denitrifying bacteria to catalyze the nitrite reduction with the lowest possible energy requirement. Since electron transfer proteins such as azurin and cytochrome c_551_ are utilized, enzymes involved in a denitrification process must receive electrons from these electron transfer proteins. For this purpose, the iron dioxo-isobacteriochlorin complex (heme d_1_), which has higher redox potential than iron porphyrin and chlorin complexes and moderate nitrite reduction activity, would be selected as the catalytic site of cytochrome cd_1_. A more electron-deficient iron center than iron dioxo-isobacteriochlorin would also not be favorable because the nitrite reduction activity is too low to function as a nitrite reductase. In addition, strong binding of NO_2_^–^ and weak binding of formed NO to iron dioxo-isobacetriochlorin complex accelerate the catalytic cycle of the nitrite reduction. Iron dioxo-isobacteriochlorin has the best balance between the redox potential, the ligand binding property, and the nitrite reduction activity. On the other hand, as proposed in the previous study, the most important point for the Cu-NiR would be how to increase the reactivity of the active site because the redox potentials of the Cu-NiR model complexes with various tridentate ligands are higher than the redox potentials of electron transfer proteins. Therefore, it appears that the Cu-NiR selects the three-His ligand environment in its reactive center because the strong electron-donating ability of the three-His ligands can increase the reactivity of the nitrite reduction without lowering the redox potential.

## Experimental section

The details of experiments are described in ESI.†

## Conclusions

To reveal the functional role of the unique heme d_1_ in the catalytic nitrite reduction, we studied effect of the porphyrin macrocycle on each reaction step of the catalytic cycle of cytochrome cd_1_ using synthetic model complexes. We show here that the dioxo-isobacteriochlorin structure is superior to porphyrin and mono-oxo-chlorin structures in the first iron reduction step, the second nitrite binding step, and the NO dissociation step, but inferior in the third nitrite reduction step. These results suggest that the heme d_1_ has evolved as the catalytic site of cytochrome cd_1_ to catalyze the nitrite reduction at the highest possible redox potential while maintaining its catalytic activity.

## Supplementary Material

Supplementary information
