# Supplementary material for: The functional role of the structure of the dioxo-isobacteriochlorin in the catalytic site of cytochrome cd1 for the reduction of nitrite
Source: Chem Sci. 2016 Jan 20;7(4):2896–906. doi: 10.1039/c5sc04825g (PMC6054029; doi:10.1039/c5sc04825g)
Supplement: Supplementary file 1 [file SC-007-C5SC04825G-s001.pdf]

Electronic Supplementary Material (ESI) for Chemical Science.
This journal is © The Royal Society of Chemistry 2016

Supplementary Information of

Functional Role of Structure of the Dioxo-isobacteriochlorin in Catalytic Site of Cytochrome cd_1 on Nitrite Reduction

Hiroshi Fujii[†,*], Daisuke Yamaki[‡], Takashi Ogura[§], Masahiko Hada[‡]

Department of Chemistry, Biology and Environmental Science, Faculty of Science, Nara Women's University, Kitauoyanishi, Nara 630-8506, Japan, Department of Chemistry, Graduate School of Science, Tokyo Metropolitan University, 1-1 Minami-Osawa, Hachioji, Tokyo 192-0397, Japan, and Department of Life Science and Picobiology Institute, Graduate School of Life Science, University of Hyogo, RSC-UH Leading Program Center, 1-1-1 Koto, Sayo-cho, Sayo-gun, Hyogo 679-5148, Japan

[*] To whom correspondence should be addressed. Tel: +81-742-20-3395

E-mail: fujii@cc.nara-wu.ac.jp

[†] Nara Women's University

[‡] Tokyo Metropolitan University

[§] University of Hyogo

General Considerations. All metal complexes were prepared and stored in a glovebox under an Ar atmosphere (H_2O and O_2 < 0.1 ppm). Anhydrous solvents were purchased from commercial sources and degassed before use. Ferric chloride complex of **3** was purchased from Tokyo Kassei. Ferric chloride complexes of **1** and **2** were synthesized as described in the literature.[1] Ferric perchlorate complex of **3** was prepared by the published method.[2] P-nitrophenolate complexes of **1** ~ **3** were shown in SI. ^{15}N-labelled PPN-$^{15}NO_2$ was synthesized from PPN-Cl and ^{15}N-labelled sodium nitrite (98 % enriched). All other reagents were obtained from commercial sources and used as received. ^{1}H NMR spectra were recorded on a JEOL JNM-LA500 spectrometer. ^{1}H NMR chemical shifts were referenced to residual solvent peaks and reported versus tetramethylsilane (TMS). UV-vis spectra were recorded on a Shimadzu Maltispec-1500 spectrometer and an Agilent 8453 spectrophotometer. Electrochemical experiments were performed using an ALS612A electrochemical analyzer (BAS) with a conventional three-electrode electrochemical cell. Tetra-*n*-butylammonium perchlorate (0.1 M) was used as a supporting electrolyte. The working and counter electrodes were platinum. The potentials were recorded with respect to a saturated calomel electrode (SCE) as a reference electrode. Spectroelectrochemistry was carried out in a custom-designed thin-layer quartz cell (optical path = 0.5 mm) with a gold-mesh (110 mesh) working electrode, a platinum-wire counter electrode, and an Ag-wire reference electrode, which were connected to a HA-151 potentiostat-galvanostat (HOKUTO DENKO). EPR spectra were recorded at room temperature on an E500 continuous-wave X-band spectrometer (Bruker). For EPR measurements at 4 K, an ESR910 helium-flow cryostat (Oxford Instruments) was used. ESI mass spectra were obtained with a LCT time-of-flight mass spectrometer equipped with an electrospray ionization interface (Micromass). Resonance Raman scattering was excited at 413.1

nm from a Kr+ laser (Spectra Physics, BeamLok 2060), dispersed by a polychromator (Ritsu Oyo Kogaku, MC-1000, focal length = 1,000 mm, 1,200 grooves / mm grating) and detected with a CCD detector (Horiba Jobin Yvon, Symphony 1024 × 128). Laser power at the sample was 4 mW. Sample concentration was 0.25 ~ 0.4 mM. A spinning cell was used at room temperature.

Chemical reduction of ferric complexes. Preparation of ferrous complexes of **1** ~ **3** was carried in a glovebox under an Ar atmosphere immediately prior to initiation of experiments. The ferric chloride complex was used as the starting complexes for **1** and ferric perchlorate complexes were used for **2** and **3**. General procedure; the ferric complex (0. 7 mg) was dissolved in acetonitrile (10 ml). A large excess of zinc powder (~ 10 mg) was added to the solution and the mixture was stirred for 10 min ~ 2 hr. The reduction process was monitored by observing absorption spectral changes. When most of the ferric complex was reduced to the ferrous state, the zinc powder was filtered off and the solution was passed through a silica gel column (1 × 5 cm) to remove remaining ferric impurities. The eluted ferrous sample was used immediately. Because of the solubility of the ferrous complex, a mixture of acetoniltrile-toluene (1:1) mixture was used as the solvent for **3**.

Quantum-chemical Calculations. Quantum-chemical calculations were performed using the Gaussian03 program package.[3] The 6-31G(d,f) basis set[4] modified by adding diffuse s- and p-type primitives was used for Fe and the 6-31G basis sets[5] were used for the other atoms. GaussView03 was used to draw orbital pictures.[6] Optimized structures of three model complexes of **1**, **2**, and **3** with imidazole and nitrite were calculated using B3LYP density functional methods. Calculations of molecular orbital energies and coefficients for drawing the orbital correlation diagram were carried out using the restricted Hartree-Fock and the restricted open-shell Hartree-Fock method.

References

1 C. K. Chang, C. Sotiriou, W. Wu, *J. Chem. Soc., Chem. Commun.* 1986, 1213-1215.

2 C. Reed, T. Mashiko, S. P. Bentley, M. E. Kastner, W. R. Scheidt, K. Spartalian, G. Lang, *J. Am. Chem. Soc.* 1979, **101**, 2948-2958.

3 Frisch, M. J.; Trucks, G. W.; Schlegel, H. B.; Scuseria, G. E.; Robb, M. A.; Cheeseman, J. R.; Montgomery, Jr., J. A.; Vreven, T.; Kudin, K. N.; Burant, J. C.; Millam, J. M.; Iyengar, S. S.; Tomasi, J.; Barone, V.; Mennucci, B.; Cossi, M.; Scalmani, G.; Rega, N.; Petersson, G. A.; Nakatsuji, H.; Hada, M.; Ehara, M.; Toyota, K.; Fukuda, R.; Hasegawa, J.; Ishida, M.; Nakajima, T.; Honda, Y.; Kitao, O.; Nakai, H.; Klene, M.; Li, X.; Knox, J. E.; Hratchian, H. P.; Cross, J. B.; Bakken, V.; Adamo, C.; Jaramillo, J.; Gomperts, R.; Stratmann, R. E.; Yazyev, O.; Austin, A. J.; Cammi, R.; Pomelli, C.; Ochterski, J. W.; Ayala, P. Y.; Morokuma, K.; Voth, G. A.; Salvador, P.; Dannenberg, J. J.; Zakrzewski, V. G.; Dapprich, S.; Daniels, A. D.; Strain, M. C.; Farkas, O.; Malick, D. K.; Rabuck, A. D.; Raghavachari, K.; Foresman, J. B.; Ortiz, J. V.; Cui, Q.; Baboul, A. G.; Clifford, S.; Cioslowski, J.; Stefanov, B. B.; Liu, G.; Liashenko, A.; Piskorz, P.; Komaromi, I.; Martin, R. L.; Fox, D. J.; Keith, T.; Al-Laham, M. A.; Peng, C. Y.; Nanayakkara, A.; Challacombe, M.; Gill, P. M. W.; Johnson, B.; Chen, W.; Wong, M. W.; Gonzalez, C.; and Pople, J. A.; Gaussian, Inc., Wallingford CT, 2004. S20.

4 V. Rassolov, J. A. Pople, M. Ratnerand, T. L. Windus, *J. Chem. Phys.* 109, 1223 (1998).

5 W. J. Hehre, R. Ditchfield and J. A. Pople, *J. Chem. Phys.* 56, 2257 (1972).

6 R., II. Dennington, T. Keith, J. Millam, K. Eppinnett, W. L. Hovell, R. Gilliland, Semichem, Inc., Shawnee Mission, KS, 2003.

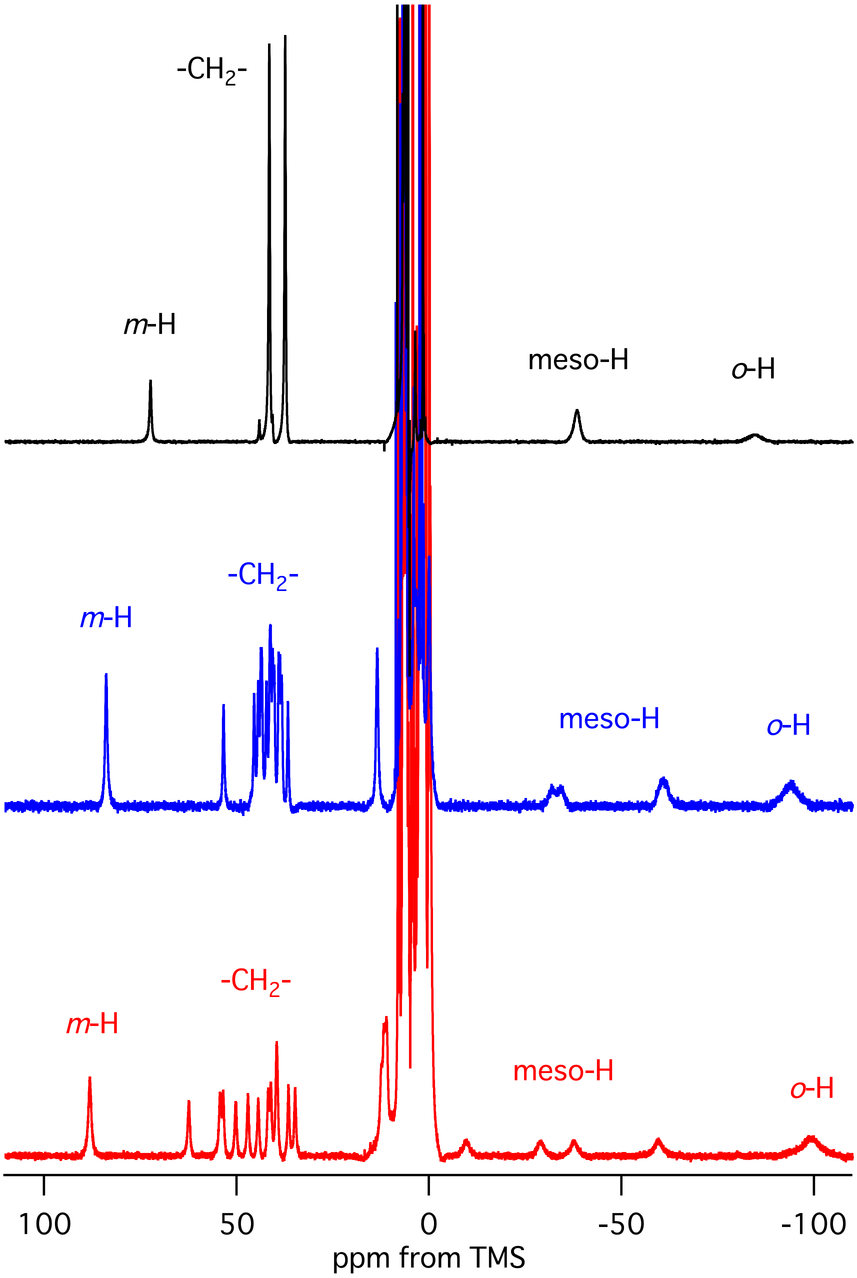

Figure S1. ^{1}H NMR spectra of *p*-nitrophenolate complexes of **1** ~ **3** in CD_2Cl_2 at 298 K. Bottom spectrum (red line): **1**, middle spectrum (blue) : **2**, top spectrum (black): **3**. The *o*- and *m*-proton proton signals of the iron-bound *p*-nitrophenolate are observed as one signal, respectively, because of faster rotation of the phenyl ring along the O–C axis than the NMR time scale. The number of the meso-H signal for 1 ~ 3 depends on the molecular symmetry of porphyrin ligand, because of free rotation of the *p*-nitrophenolate group along the Fe–O axis, four meso-H signals for **1** and **2** and one meso-H signal for **3**. The assignments of the *o*- and *m*-signals are based on the spin polarization mechanism from ferric high-spin iron.

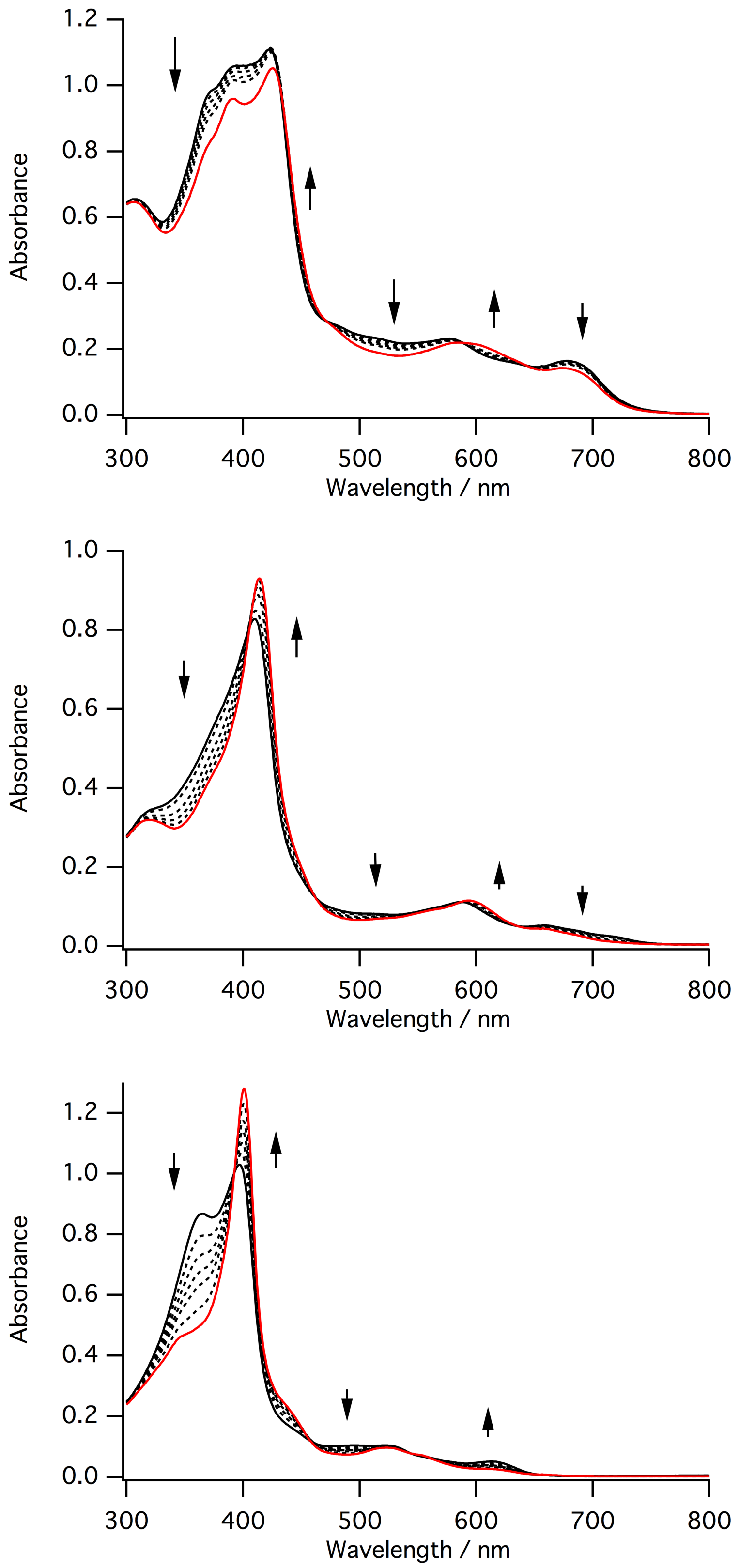

Figure S2. Absorption spectral changes for the binding of 1-methylimidazole to 1-methylimidazole and p-nitrophenolate mixed ligand complexes of **1** ~ **3**. Top: **1**, middel: **2**, bottom: **3**. Black lines: the mixed ligand complexes, red line: bis-1-methylimidazole complex.

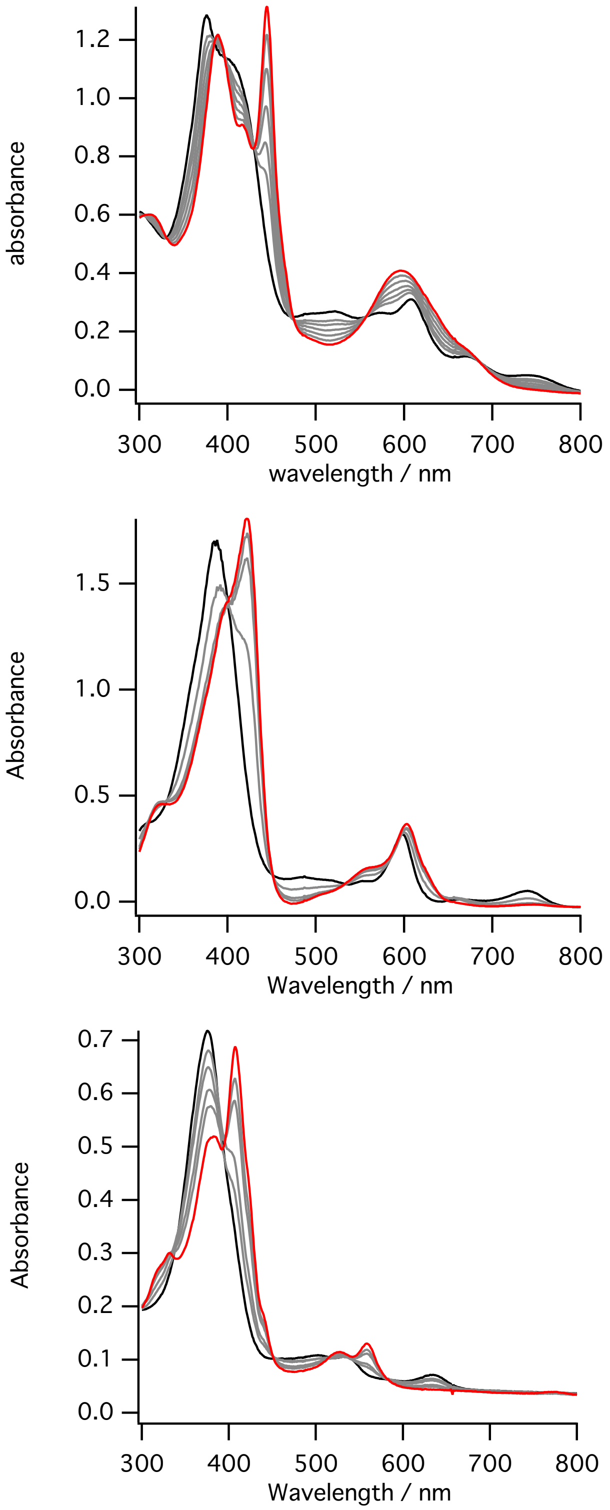

Figure S3. Absorption spectral change for electrochemical reduction of ferric chloride complexes of **1** (top), **2** (middle) and **3** (bottom) in acetonitrile containing 0.1 M tetra-n-butylammonium perchlorate under anaerobic conditions at 298 K. The applied voltages were 100 mV ~ 300 mV lower than the $E_{1/2}$ values for ferric/ferrous redox couples.

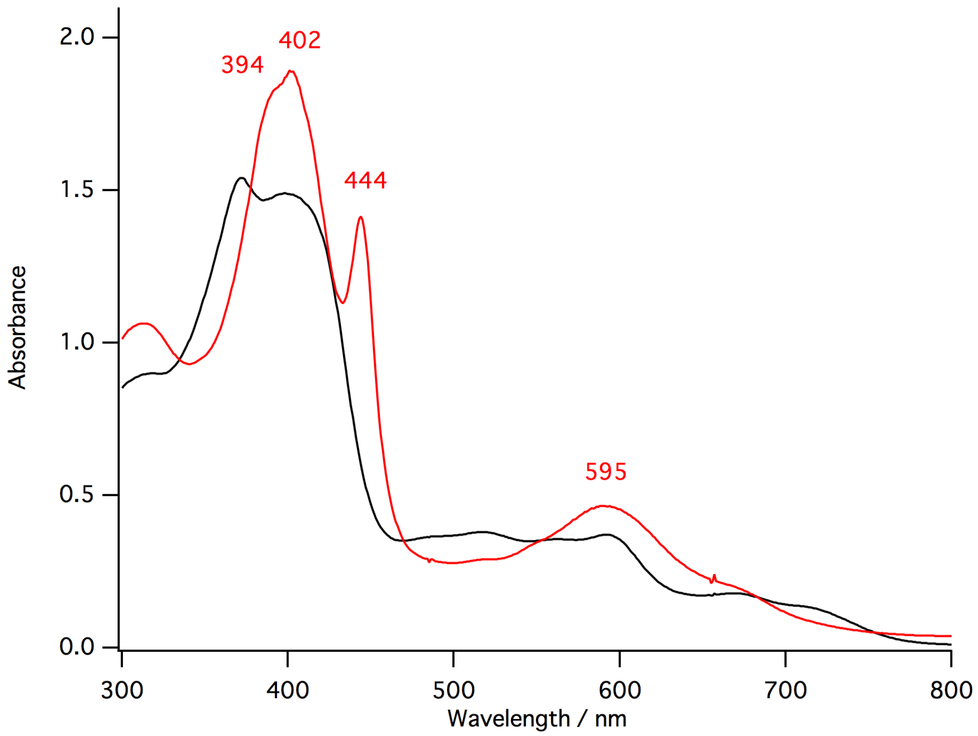

Figure S4. Absorption spectra of ferric p-nitrophenolate complex of **1** (black line) and its reduced complex with zinc powder (red line) in acetonitrile. The absorption spectrum of the ferrous complex prepared from the ferric p-nitrophenolate complex of **1** is close to that of the ferrous complex prepared from ferric chloride complex of **1** (Figure S3 top). Because of the overlap of the absorption peak of the released *p*-nitrophenolate anion, the intensity of the Soret band at 394 nm becomes unclear.

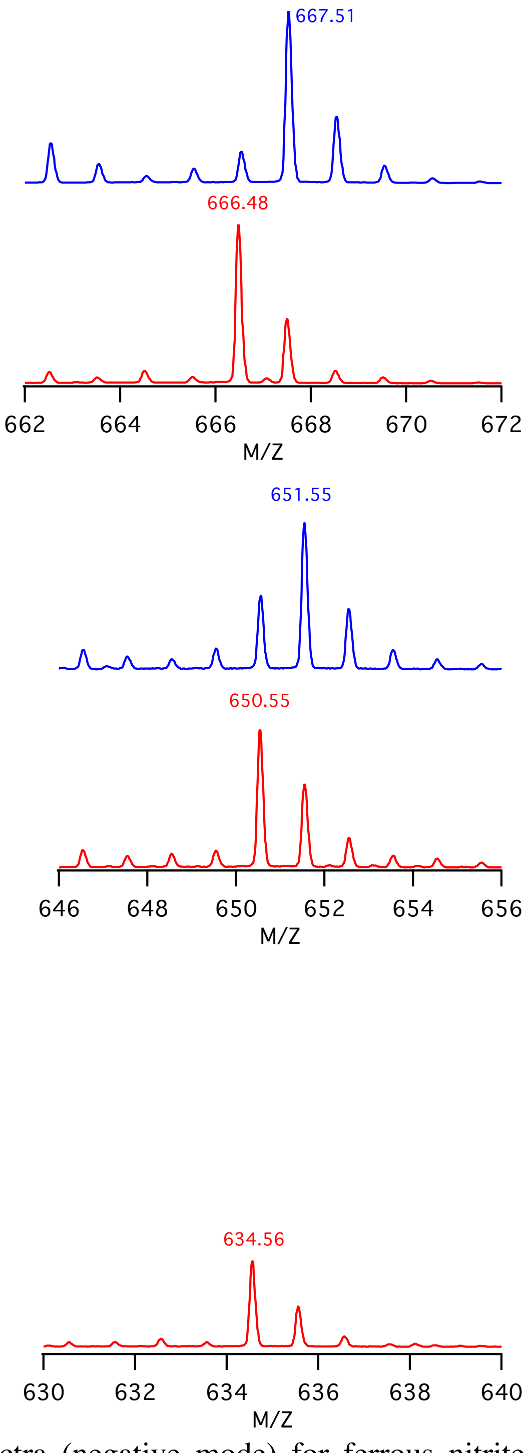

Figure S5. ESI-MS spectra (negative mode) for ferrous nitrite complexes of **1** (top), **2** (middle), and **3** (bottom). Blue lines show ESI-MS spectra for ^{15}N-labelled ferrous nitrite complexes of **1** ~ **3**. The ^{15}N-labelled samples for ESI-MS measurements were prepared from ^{15}N-labelled sodium nitrite ($Na^{15}NO_2$) dissolved in methanol, instead of PPN-$^{15}NO_2$. Because of instability of the sample, we could not get ESI-MS spectrum for ^{15}N-labelled sample of **3**.

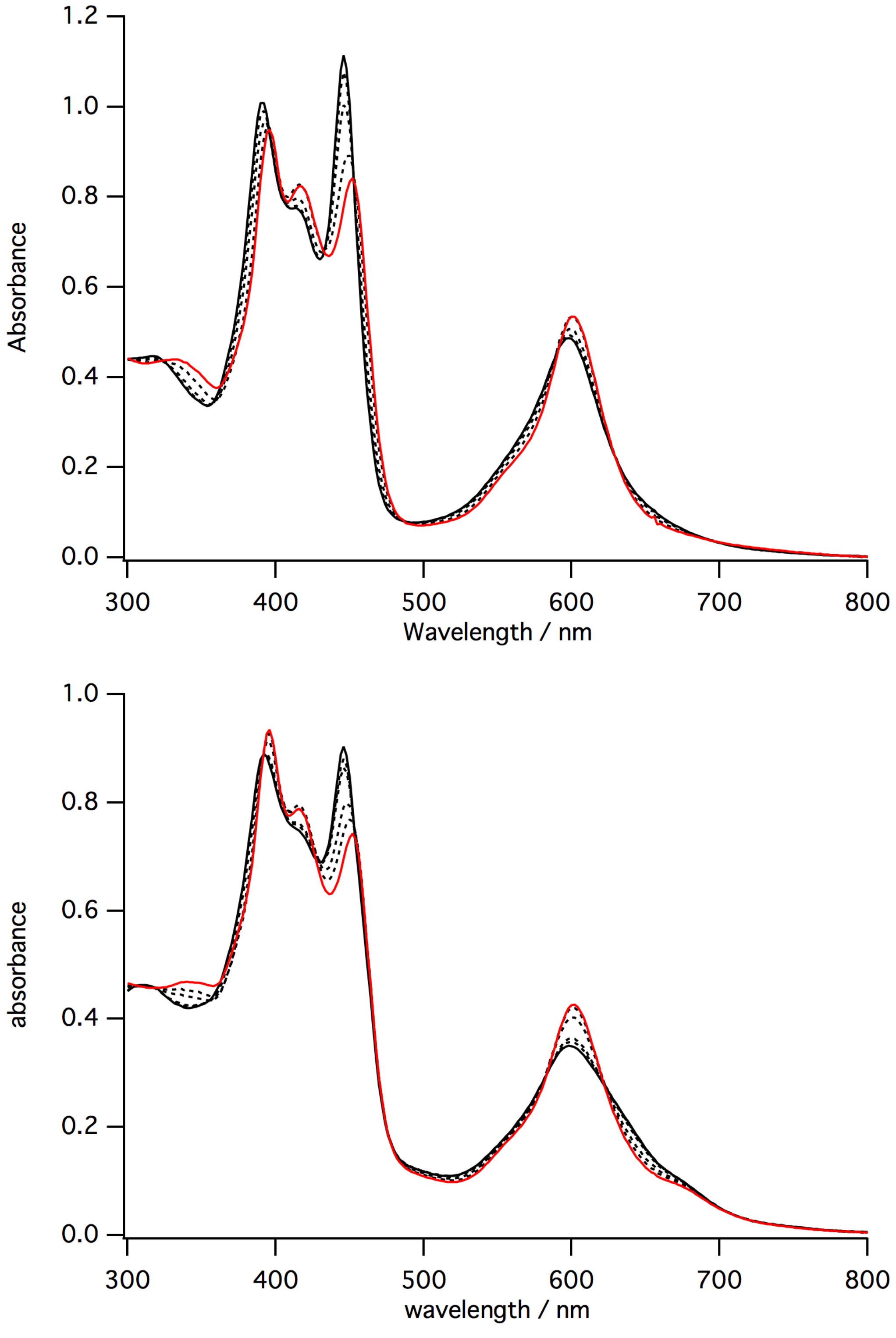

Figure S6. Absorption spectral change for the titration of ferrous complexes of **1** with PPN-NO_2 in the presence of 1-methylimidazole (top: 1-equiv) and 1,2-dimethylimidazole (bottom: 50-equiv) in acetonitrile at 298K. The black and red lines show ferrous complexes and ferrous nitrite complexes in the presence of imidazole ligands, respectively.

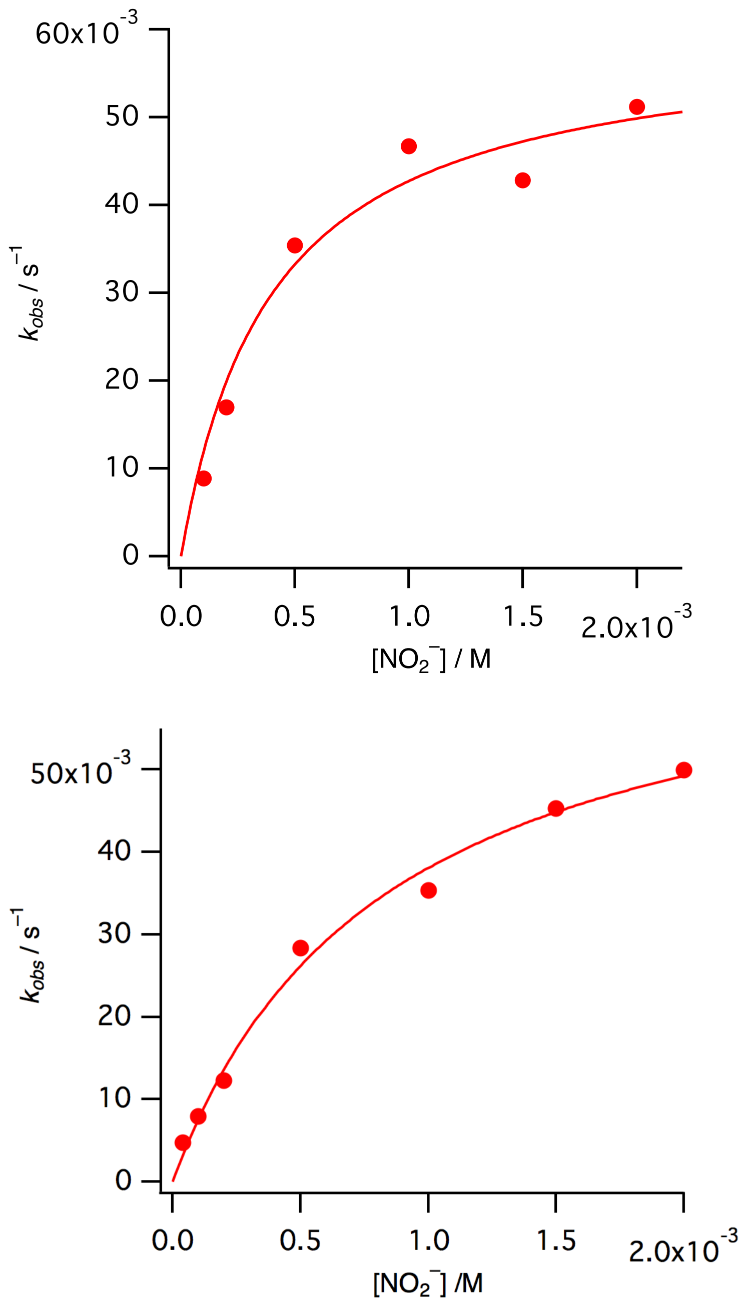

Figure S7. Dependence of apparent reaction rate constant on the concentration of nitrite at the constant concentration of acetic acid in acetonitrile at 298 K. Top: **1** at [CH_3COOH] = 6.0 mM and bottom: **2** at [CH_3COOH] = 1.0 mM. The estimated *c* and K values are 6.2×10^{-2} s^{-1} and 2.2×10^{3} M^{-1} for **1** and 7.0×10^{-2} s^{-1} and 1.2×10^{3} M^{-1} for **2**, respectively.

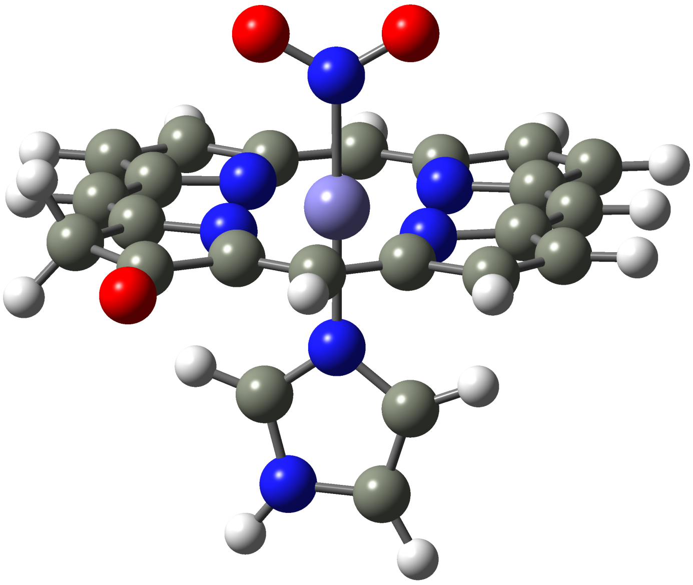

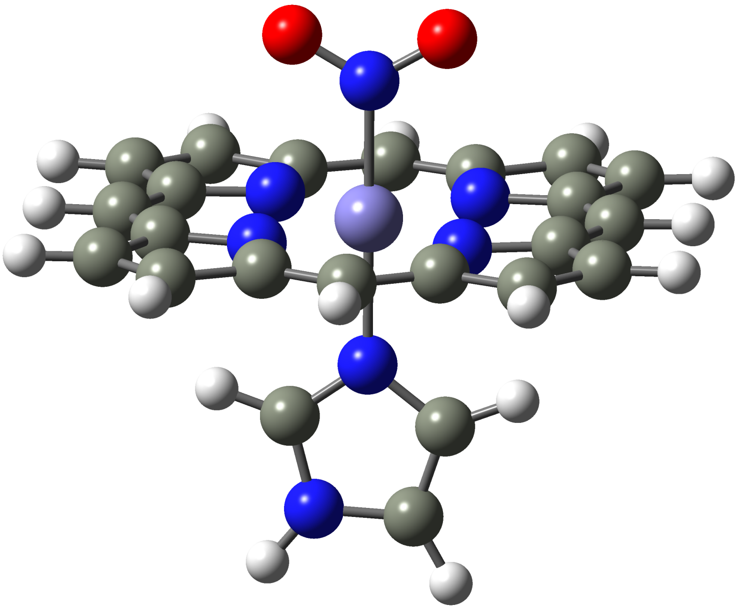

Figure S8. Structures of **2** and **3** used in the present molecular orbital calculations.

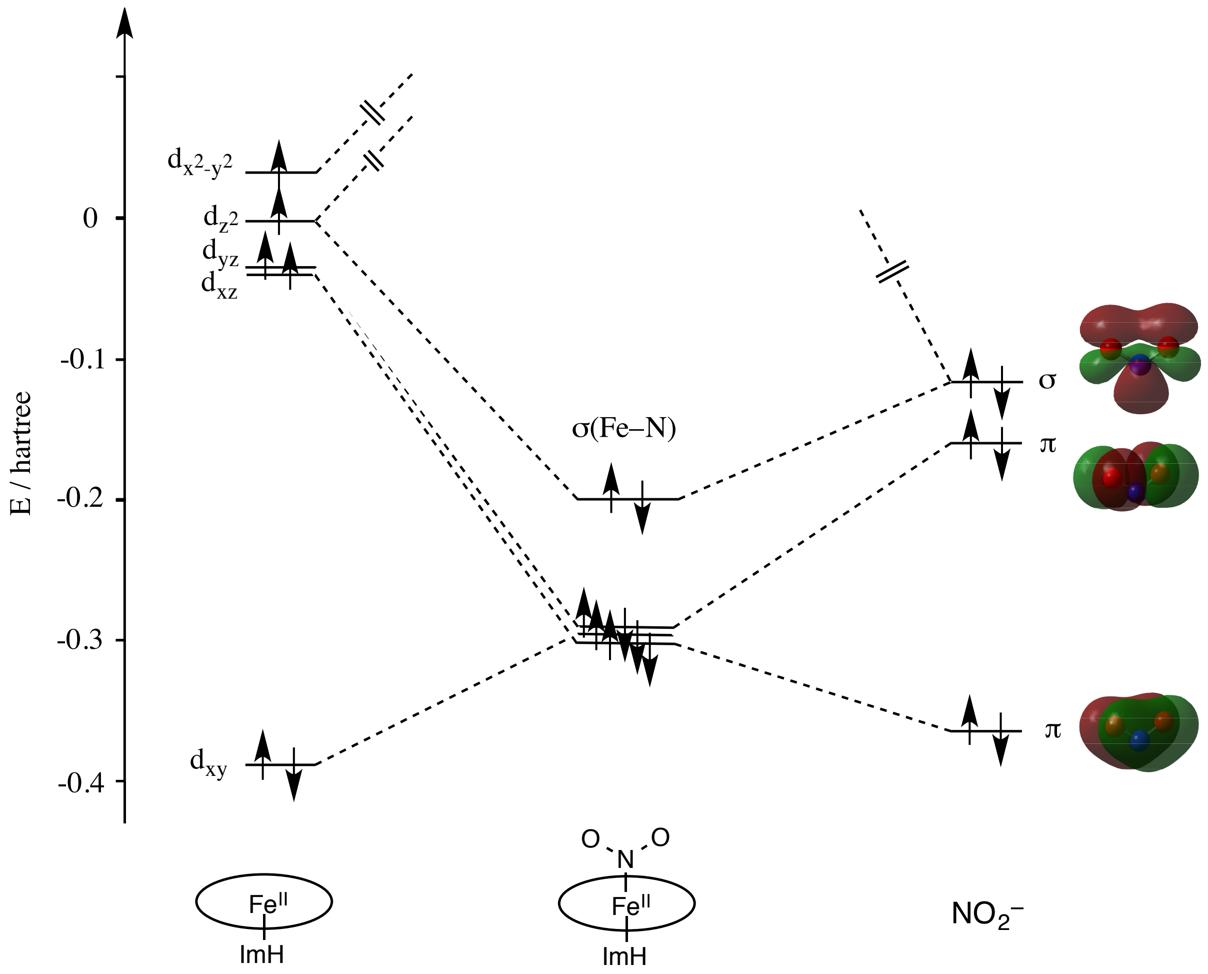

Figure S9. Molecular orbital diagram of ferrous nitrite complex of **1**.

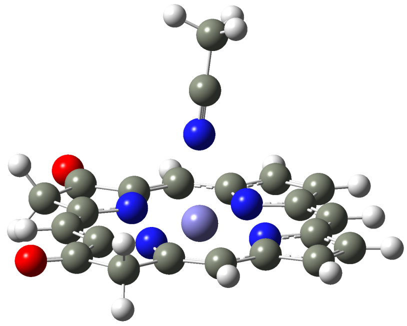

1

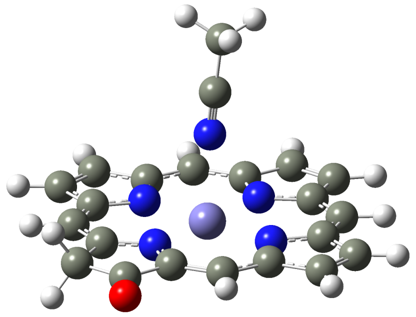

2

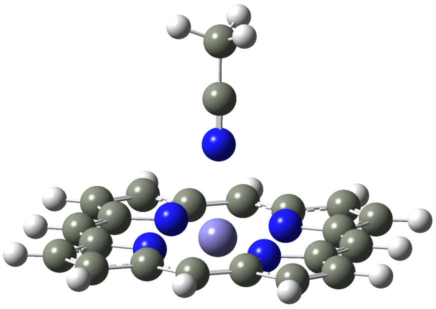

3

Figure S10. Optimized structures of ferrous mono-acetonitrile complexes of **1** ~ **3**.

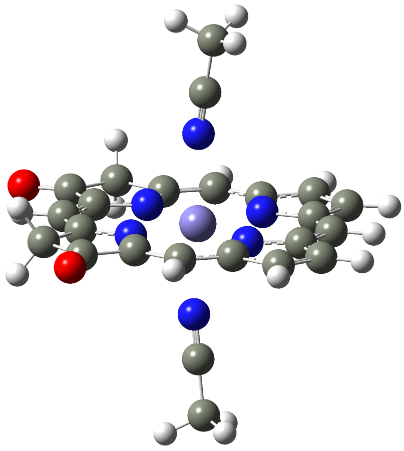

1

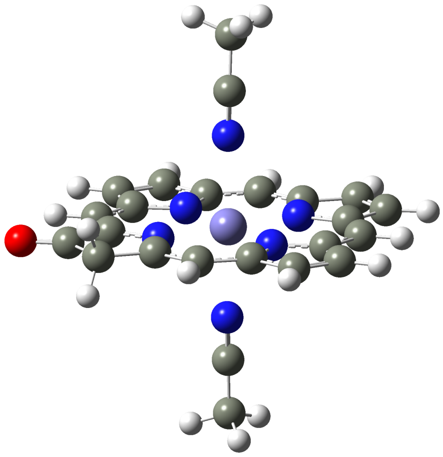

2

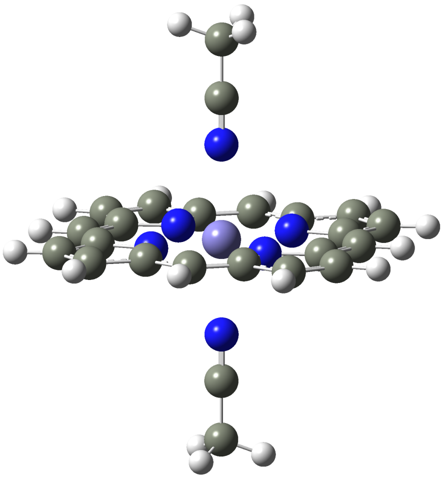

3

Figure S11. Optimized structures of ferrous bis-acetonitrile complexes of **1** ~ **3**.

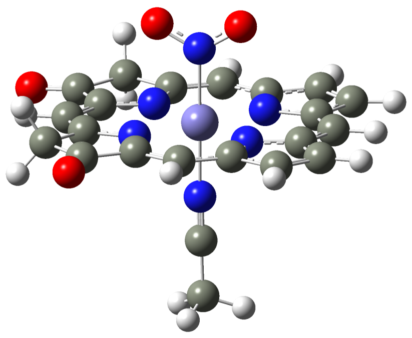

1

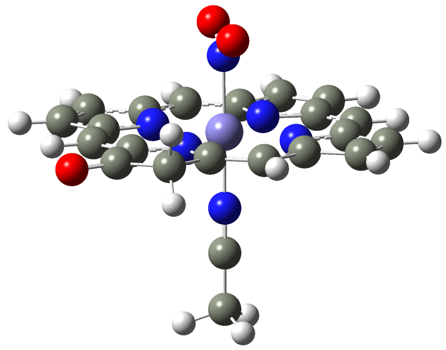

2

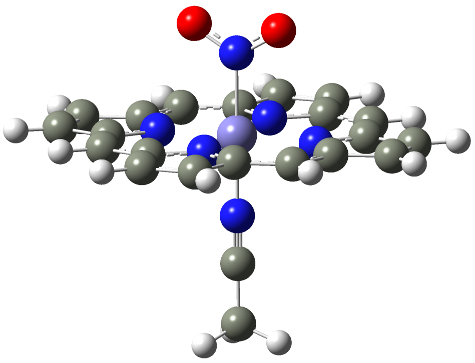

3

Figure S12. Optimized structures of ferrous mono-acetonitrile and nitrite complexes of **1** ~ **3**.

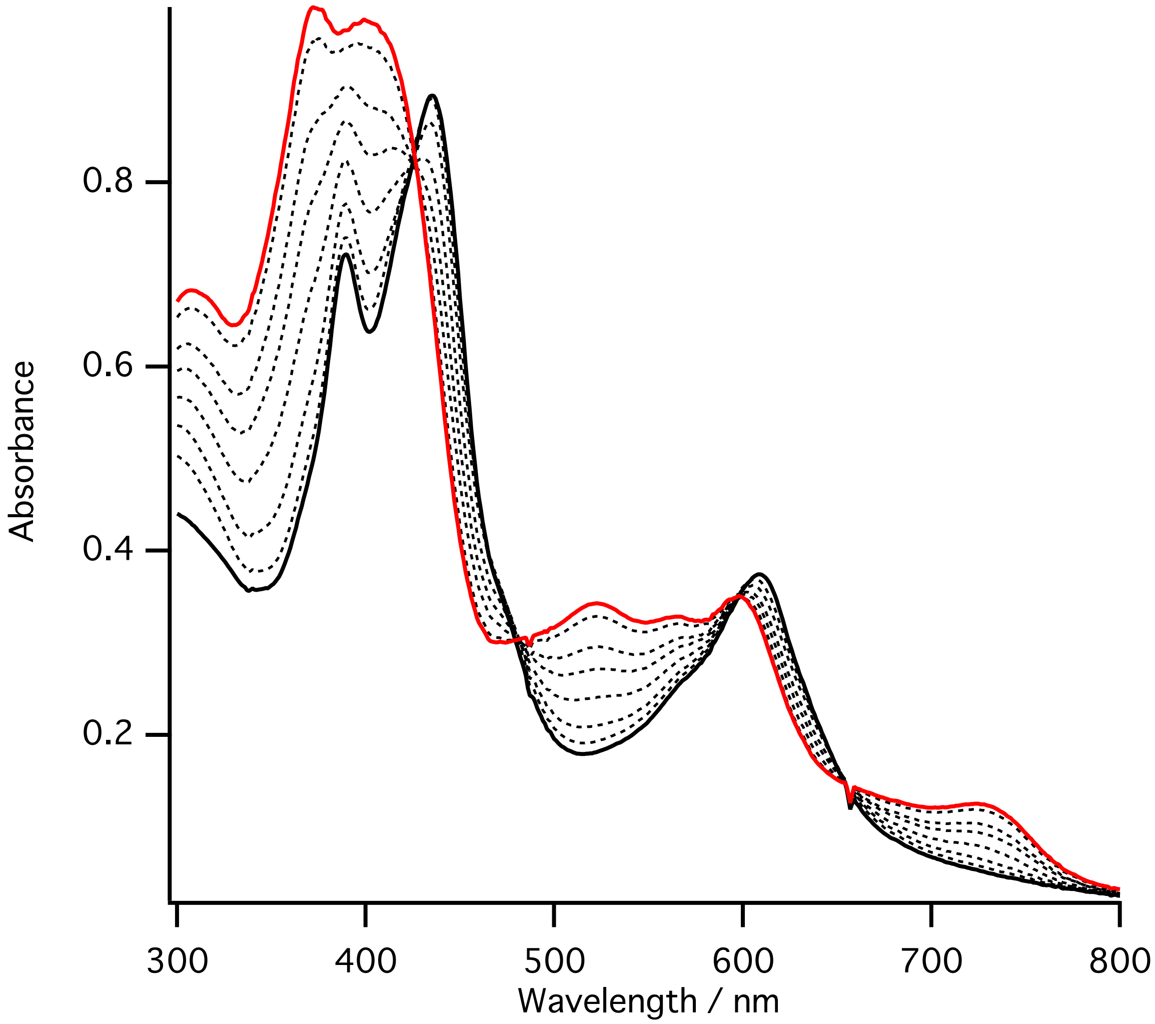

Figure S13. Absorption spectral change for the titration of ferric 1-methylimidazole-nitric oxide complex of **1** with tetrabutylammonium *p*-nitrophenolate. The initial 1-methylimidazole-nitric oxide complex of **1** was shown with black line and the spectrum after addition of 1 equiv of tetrabutylammonium *p*-nitrophenolate is shown with red line. The final spectrum was close to that of ferric *p*-nitrophenolate complex of **1**.

Table S1. Specific geometrical parameters of the structurally simplified model structures for **1**, **2** and **3**.

	**1**	**2**	**3**	unit
Fe-N(NO_2)	1.960	1.963	1.965	Å
Fe-N(Im)	2.090	2.091	2.094	Å
N-O in NO_2	1.243	1.245	1.247	Å
Fe-N1 (porphyrin)	2.039	2.019	2.020	Å
Fe-N2 (porphyrin)	2.030	2.029	2.020	Å
Fe-N3 (porphyrin)	2.042	2.033	2.019	Å
Fe-N4 (porphyrin)	2.033	2.026	2.019	Å
O-C in aldehyde	--	1.226	1.223, 1.226	Å
∠ONO in NO_2	120.0	119.8	119.6	degree

Table S2. SCF Energies (hartree) and calculated bonding energies of Fe-PorX + NO_2.

	**1**	**2**	**3**	unit[5]
Fe-PorX[1]				
Quintet[2], A	-2619.05419	-2544.18542	-2469.31924	hartree
Singlet[3], B	-2618.97842	2544.11214	-2469.24511	hartree
$E_B - E_A$	0.07577	0.07328	0.07413	hartree
Fe-PorX-NO_2, C	-2823.08155	-2748.20516	-2673.32777	hartree
NO_2[1], D	-204.06053	-204.06049	-204.06045	hartree
Binding energy [4]				
$E_B + E_D - E_C$	58.2	85.4	112.2	kJ/mol
$\Delta(E_B + E_D - E_C)$	-54.0	-26.8	0	kJ/mol

(1) These molecular geometries are taken from the optimized molecular geometries of Fe-PorX-NO_2.

(2) Electron-configuration is $(d_{xy})^2(d_{xz})^1(d_{yz})^1(d_{z2})^1(d_{x2-y2})^1$ of Fe(II)

(3) Electron-configuration is $(d_{xy})^2(d_{xz})^2(d_{yz})^2(d_{z2})^0(d_{x2-y2})^0$ of Fe(II)

(4) The estimation of the relative stability between the singlet and quintet states is difficult, since the open-shell SCF calculation with high-spin-multiplicity inevitably includes the electron-correlation energy. Therefore all binding energies calculated by E(A) + E(D) – E(C) are negative, and this means that nitride complexes C are unstable in our MO calculations. So we used the energies of singlet states and calculated the binding energy by $E_B + E_D - E_C$.

(5) 1 hartree = 627.5095 kcal/mol

Table S3. Occupied orbital energies (hartree)[2] of **1**, **2**, and **3** and their character by the open-shell restricted Hartree-Fock (ROHF) method.

**1**			**2**			**3**		
#	energy	character	#	energy	character	#	energy	character
121	0.01157[1]	$3d_{x2-y2}$(Fe)	117	0.02044[1]	3dδ(Fe)	113	0.03445[1]	3dδ(Fe)
120	-0.04993	$3d_{z2}$(Fe)	116	-0.03840	$3d_{z2}$(Fe)	112	-0.03091	$3d_{z2}$(Fe)
119	-0.05835	$3d_{xz}$(Fe)	115	-0.04448	$3d_{xz}$(Fe)	111	-0.03878	$3d_{xz}$(Fe)
118	-0.06027	$3d_{yz}$(Fe)	114	-0.05015	$3d_{yz}$(Fe)	110	-0.04049	$3d_{yz}$(Fe)
117	-0.20860	π(Por)	113	-0.21011	π(Por)	109	-0.20154	π(Por)
116	-0.25741	π(Por)	112	-0.24418	π(Por)	108	-0.22420	π(Por)
115	-0.32721	π(Por)	111	-0.32599	Im	107	-0.31638	Im+π(Por)
114	-0.33620	Im	110	-0.32862	π(Por)+Im	106	-0.32831	$3d_{xy}$(Fe)
113	-0.34025	π(Por)	109	-0.33668	π(Por)	105	-0.33326	π(Por)
112	-0.35725	π(Por)	108	-0.34498	π(Por)	104	-0.33348	π(Por)+Im
111	-0.35837	π(Por)	107	-0.35626	π(Por)	103	-0.34220	π(Por)
110	-0.39699	π*(O=C)	106	-0.35781	π(Por)	102	-0.35459	π(Por)
109	-0.41337	$3d_{xy}$(Fe)	105	-0.39597	π*(O=C)	101	-0.35569	π(Por)
108	-0.41710	π*(O=C)	104	-0.40046	$3d_{xy}$(Fe)	100	-0.38317	$3d_{xy}$(Fe)

(1) HOMO

(2) 1 hartree = 627.5095 kcal/mol

Table S4. Orbital energies (hartree)[2] of **1**, **2**, and **3** with NO_2^- and their character by the closed-shell restricted Hartree-Fock (RHF) method.

**1** with NO_2^-			**2** with NO_2^-			**3** with NO_2^-		
#	energy	character	#	energy	character	#	energy	character
131	-0.09774[1]	π(Por)	127	-0.09522[1]	π(Por)	123	-0.09089[1]	π(Por)
130	-0.13457	π(Por)	126	-0.12005	π(Por)	122	-0.10504	π(Por)
129	-0.20724	π(Por)	125	-0.20437	π_n (NO_2)	121	-0.19762	π_n (NO_2)
128	-0.21549	σ(Fe-N)	124	-0.20744	σ(Fe-N)	120	-0.19817	σ(Fe-N)
127	-0.22154	σ(Fe-N)	123	-0.20930	σ(Fe-N)+Porπ	119	-0.20130	π(Por)
126	-0.22441	π_n (NO_2)	122	-0.21439	π_n (NO_2)	118	-0.2017	π(Por)
125	-0.24180	Im	121	-0.23024	Im	117	-0.21930	Im+Porπ
124	-0.24464	Im+π(Por)+σ(NO_2)	120	-0.23739	Im+π(Por)+σ(NO_2)	116	-0.22835	π(Por)+σ(NO_2)
123	-0.24697	π(Por)	119	-0.23834	Im+π(Por)+σ(NO_2)	115	-0.22991	π(Por)
122	-0.25628	σ_n(NO_2)	118	-0.24669	π(Por)	114	-0.23111	Im
121	-0.29274	π(C=O)	117	-0.25118	π(Por)+π(NO_2)	113	-0.24691	Por-π
120	-0.30540	$3d_{xz}$(Fe)+Im	116	-0.29110	π(C=O)	112	-0.24724	Por-π
119	-0.30888	π(Por)	115	-0.29647	$3d_{xz}$(Fe)+Im	111	-0.28640	$3d_{xz}$(Fe)+Im
118	-0.31093	π(C=O)	114	-0.30548	$3d_{xy}$(Fe)	110	-0.29106	$3d_{xy}$(Fe)
117	-0.32009	$3d_{xy}$(Fe)	113	-0.30728	$3d_{yz}$(Fe)+π(NO_2)	109	-0.29849	$3d_{yz}$(Fe)+π(NO_2)
116	-0.32029	$3d_{yz}$(Fe)+π(NO_2)	112	-0.30913	$3d_{yz}$(Fe)+π(NO_2)	108	-0.30502	π(Por)
115	-0.32847	$3d_{xy}$(Fe)+Im	111	-0.32396	$3d_{xy}$(Fe)+Im	107	-0.31570	$3d_{xy}$(Fe)+Im

(1) HOMO

(2) 1 hartree = 627.5095 kcal/mol

Table S5. Orbital energies (hartree)[2] of NO_2^- and their character by the closed-shell restricted Hartree-Fock (RHF) method.

#	energy	character
13	0.42430	π*
12	-0.11518[1]	σ(lone-pair on N)
11	-0.15757	π_n (non-bondong)
10	-0.18553	σ_n (non-bondong)
9	-0.36050	π_b (bondong)
8	-0.39880	σ_b (bondong)

(1) HOMO

(2) 1 hartree = 627.5095 kcal/mol

Table S6. SCF Energies (hartree) of ferrous compleses and ferrous nitrite complexes of **1** ~ **3**.

	**1**	**2**	**3**
(P)$Fe^{II}(CH_3CN)_2$	-2668.02424798	-2592.80705216	-2517.57661875
(P)$Fe^{II}(CH_3CN)$	-2535.27472339	-2460.05212707	-2384.82386751
(P)$Fe^{II}(NO_2)(CH_3CN)$	-2740.46713137	-2665.23403616	-2589.99747429
(P)$Fe^{II}(NO_2)$	-2607.70508420	-2532.47427314	-2457.23901767
NO_2^-	-205.114101186		
CH_3CN	-132.751485923		

Table S7. The binding energies of nitrite to ferrous acetonitrile complexes of **1** ~ **3**.

$(P)Fe^{II}(CH_3CN)_2 + NO_2^- \rightarrow (P)Fe^{II}(NO_2)(CH_3CN) + CH_3CN$	
**1**	-50.4 kcal/mol
**2**	-40.4 kcal/mol
**3**	-36.5 kcal/mol
$(P)Fe^{II}(CH_3CN) + NO_2^- \rightarrow (P)Fe^{II}(NO_2)(CH_3CN)$	
**1**	-49.1 kcal/mol
**2**	-42.5 kcal/mol
**3**	-37.3 kcal/mol
$(P)Fe^{II}(CH_3CN)_2 + NO_2^- \rightarrow (P)Fe^{II}(NO_2) + 2CH_3CN$	
**1**	-43.7 kcal/mol
**2**	-35.2 kcal/mol
**3**	-32.2 kcal/mol
$(P)Fe^{II}(CH_3CN) + NO_2^- \rightarrow (P)Fe^{II}(NO_2) + CH_3CN$	
**1**	-42.5 kcal/mol
**2**	-37.4 kcal/mol
**3**	-33.0 kcal/mol

Table S8. Orbital energies (hartree)[(2)] of the $3d_{z^2}$ orbitals of ferrous complexes and the HOMO of ferrous nitrite complexes calculated from the closed-shell restricted Hartree-Fock (RHF) method

	**1**	**2**	**3**
$3d_{z^2}$ orbitals of $(P)Fe^{II}(CH_3CN)_2$	-0.25590	-0.24614	-0.22455
$3d_{z^2}$ orbitals of $(P)Fe^{II}(CH_3CN)$	-0.29050	-0.27640	-0.27060
The HOMO of $(P)Fe^{II}(NO_2)(CH_3CN)$	-0.32348	-0.31670	-0.27648

Derivations of Eq. 4 ~ 6 from Eq. 1 ~ 3.

From Eq. 1,

$$K = \frac{[(P)Fe\text{-}NO_2^-]}{[(P)Fe][NO_2^-]}$$

$$[(P)Fe] + [(P)Fe - NO_2^-] = \frac{[(P)Fe - NO_2^-]}{K[NO_2^-]} + [(P)Fe - NO_2^-]$$

$$= (\frac{1}{K[NO_2^-]} + 1)[(P)Fe - NO_2^-]$$

When concentration of nitrite is large excess and constant, $\left(\frac{1}{K[NO_2^-]} + 1\right) = constant$ and $\frac{[(P)Fe\text{-}NO_2^-]}{[(P)Fe]} = constant.$

Thus, the change of [(P)Fe] + [(P)Fe–NO_2^-] can be represented with the change of [(P)Fe–NO_2^-] and we do not have to consider about the equilibrium Eq.1 for kinetic analysis. The absorption spectral change should have isosbestic points when the first protonation step is the rate-limiting step: [(P)Fe–$NO_2H(CH_3CO_2)$] ≈ 0.

From Eq. 2 and 3,

$$-\frac{d[(P)Fe\text{-}NO_2^-]}{dt} = k_2[(P)Fe\text{-}NO_2^-][CH_3COOH] - k_{-2}[(P)Fe\text{-}NO_2H(CH_3CO_2)]$$

$$-\frac{d[(P)Fe\text{-}NO_2H(CH_3CO_2)]}{dt}$$
$$= k_3[(P)Fe\text{-}NO_2H(CH_3CO_2)][CH_3CO_2H] + k_{-2}[(P)Fe\text{-}NO_2H(CH_3CO_2)]$$
$$- k_2[(P)Fe\text{-}NO_2^-][CH_3CO_2H]$$

Here, when we introduce the steady state approximation for [(P)Fe–$NO_2H(CH_3CO_2)$],

$$-\frac{d[(P)Fe\text{-}NO_2H(CH_3CO_2)]}{dt}$$
$$= k_3[(P)Fe\text{-}NO_2H(CH_3CO_2)][CH_3CO_2H] + k_{-2}[(P)Fe\text{-}NO_2H(CH_3CO_2)]$$
$$- k_2[(P)Fe\text{-}NO_2^-][CH_3CO_2H] = 0$$

$$(k_3[CH_3CO_2H] + k_{-2})[(P)Fe\text{-}NO_2H(CH_3CO_2)] = k_2[(P)Fe\text{-}NO_2^-][CH_3CO_2H]$$

$$[(P)Fe\text{-}NO_2H(CH_3CO_2)] = k_2\frac{[(P)Fe\text{-}NO_2][CH_3CO_2H]}{(k_3[CH_3CO_2H] + k_{-2})}$$

Thus,

$$-\frac{d[(P)Fe\text{-}NO_2^-]}{dt} = k_2[(P)Fe\text{-}NO_2^-][CH_3COOH] - k_2k_{-2}\frac{[(P)Fe\text{-}NO_2^-][CH_3CO_2H]}{k_3[CH_3CO_2H] + k_{-2}}$$

$$= \frac{k_2k_3[(P)Fe\text{-}NO_2^-][CH_3COOH]^2}{k_3[CH_3CO_2H] + k_{-2}}$$

$$= \frac{k_2}{\frac{k_{-2}}{k_3} + [CH_3CO_2H]}[(P)Fe\text{-}NO_2^-][CH_3COOH]^2 \quad (\text{Eq. }4)$$

Under excess acetic acid concentrations (the pseudo-first order condition) and constant nitrite concentrations (i.e., the initial concentrations of ferrous nitrite complex is constant),

$$k_{obs} = \frac{k_2[CH_3COOH]^2}{\frac{k_{-2}}{k_3} + [CH_3CO_2H]} \quad (\text{Eq. }6)$$

On the other hand, when the concentration of nitrite is large excess and the concentration of acetic acid is large excess and constant,

$$-\frac{d\{[(P)Fe\text{-}NO_2^-] + [(P)Fe]\}}{dt} = -(\frac{1}{K[NO_2^-]} + 1)\frac{d[(P)Fe\text{-}NO_2^-]}{dt} = c[(P)Fe\text{-}NO_2^-]$$

$$-\frac{d[(P)Fe\text{-}NO_2^-]}{dt} = c(\frac{K[NO_2^-]}{1 + K[NO_2^-]})[(P)Fe\text{-}NO_2^-]$$

$$k_{obs} = c(\frac{K[NO_2^-]}{1 + K[NO_2^-]})$$
